# The *Fusarium graminearum* Histone H3 K27 Methyltransferase KMT6 Regulates Development and Expression of Secondary Metabolite Gene Clusters

**DOI:** 10.1371/journal.pgen.1003916

**Published:** 2013-10-31

**Authors:** Lanelle R. Connolly, Kristina M. Smith, Michael Freitag

**Affiliations:** Department of Biochemistry and Biophysics, Center for Genome Research and Biocomputing, Oregon State University, Corvallis, Oregon, United States of America; University of California San Francisco, United States of America

## Abstract

The cereal pathogen *Fusarium graminearum* produces secondary metabolites toxic to humans and animals, yet coordinated transcriptional regulation of gene clusters remains largely a mystery. By chromatin immunoprecipitation and high-throughput DNA sequencing (ChIP-seq) we found that regions with secondary metabolite clusters are enriched for trimethylated histone H3 lysine 27 (H3K27me3), a histone modification associated with gene silencing. H3K27me3 was found predominantly in regions that lack synteny with other *Fusarium* species, generally subtelomeric regions. Di- or trimethylated H3K4 (H3K4me2/3), two modifications associated with gene activity, and H3K27me3 are predominantly found in mutually exclusive regions of the genome. To find functions for H3K27me3, we deleted the gene for the putative H3K27 methyltransferase, KMT6, a homolog of *Drosophila* Enhancer of zeste, E(z). The *kmt6* mutant lacks H3K27me3, as shown by western blot and ChIP-seq, displays growth defects, is sterile, and constitutively expresses genes for mycotoxins, pigments and other secondary metabolites. Transcriptome analyses showed that 75% of 4,449 silent genes are enriched for H3K27me3. A subset of genes that were enriched for H3K27me3 in WT gained H3K4me2/3 in *kmt6*. A largely overlapping set of genes showed increased expression in *kmt6*. Almost 95% of the remaining 2,720 annotated silent genes showed no enrichment for either H3K27me3 or H3K4me2/3 in *kmt6*. In these cases mere absence of H3K27me3 was insufficient for expression, which suggests that additional changes are required to activate genes. Taken together, we show that absence of H3K27me3 allowed expression of an additional 14% of the genome, resulting in derepression of genes predominantly involved in secondary metabolite pathways and other species-specific functions, including putative secreted pathogenicity factors. Results from this study provide the framework for novel targeted strategies to control the “cryptic genome”, specifically secondary metabolite expression.

## Author Summary

Changes in chromatin structure are required for time- and tissue-specific gene regulation. How exactly these changes are mediated is under intense scrutiny. The interplay between activating histone modifications, e.g. H3K4me, and the silencing H3K27me3 mark has been recognized as critical to orchestrate differentiation and development in plants and animals. Here we show that filamentous fungi, exemplified by the cereal pathogen *Fusarium graminearum*, can use H3K27 methylation to generate silenced, facultative heterochromatin, covering more than a third of the genome, much more than the 5–8% of *Neurospora* or metazoan genomes. Removal of the silencing mark by mutation of the methyltransferase subunit of the PRC2 silencing complex resulted in activation of more than 1,500 genes, 14% of the genome. We show that generation of facultative heterochromatin by H3K27 methylation is an ancestral process that has been lost in certain lineages (e.g. at least some hemiascomycetes, the genus *Aspergillus* and some basidiomycetes). Our studies will open the door to future precise “epigenetic engineering” of gene clusters that generate bioactive compounds, e.g. putative mycotoxins, antibiotics and industrial feedstocks. Availability of tractable fungal model systems for studies of the control and function of H3K27 methylation may accelerate mechanistic research.

## Introduction

Histone lysine methylation provides an epigenetic layer for transcriptional regulation, with particular methylation sites associated with active (H3K4me2/3) or repressive (H3K9me2/3 and H3K27me2/3) regions of chromatin [1]. Polycomb group (PcG) transcriptional repressors that generate and read the H3K27me3 mark were first genetically identified in *Drosophila* as negative regulators of Hox developmental genes [2]; they repress many additional developmental regulators by generating “facultative heterochromatin” [3],[4],[5]. Certain genes can be associated with both activating (e.g., H3K4me2/3) and silencing (e.g. H3K27me3) marks, and thus form “bivalent domains” [6] that are thought to be metastable and poised for either repression or activation during differentiation and development. The precise location of the two marks across genes is different, however, as H3K4me3 is found at the transcriptional start site (TSS) or directly downstream of it, while H3K27me3 is found both up- and downstream of the H3K4me3 peaks [7].

PcG repressive marks are opposed by activating H3K4me marks that are established by Trithorax group (TrxG) proteins in *Drosophila* [8]. In human [9],[10], *Arabidopsis* [11],[12], and yeast [13],[14], active gene promoters are associated with H3K4me3, and H3K4me2 serves as an epigenetic memory of prior transcription. H3K36me3 methyltransferases are associated with elongating RNA polymerase and generate this mark at the 3′ end of transcribed genes [15].

With this study we are beginning to uncover an important physiological role for PcG proteins in fungi. Previously, H3K27me3 had been detected in *Neurospora crassa* [16] but its function in this species remains unclear [17]. In our studies on centromeres of the cereal pathogen *Fusarium graminearum* (teleomorph: *Gibberella zeae*), we found that H3K27me3 was absent from pericentric regions, similar to what has been found in plants [18],[19]. By chromatin immunoprecipitation (ChIP) followed by high-throughput DNA sequencing (ChIP-seq), we found that extensive segments, covering a third of the genome, were enriched with H3K27me3 in *F. graminearum*. Here we show that a *Fusarium* homologue of the *Drosophila* H3K27 methyltransferase Enhancer of zeste [E(z)], an enzyme we call KMT6 in accordance with proposed nomenclature [20], generates H3K27me3 marks that cover 46% of all genes. As expected, a majority of these genes is not expressed in wildtype cells, while in the absence of KMT6 an additional 14% of all genes were induced. These were predominantly genes involved in the production or detoxification of secondary metabolites or predicted to play a role in pathogenicity. We provide a chromatin-based model for coordinated expression of many gene clusters predominantly located in extant or ancestral subtelomeric regions and containing species-specific genes of unknown function.

## Results

### The Pcg Complement Of Filamentous Fungi

We searched the currently available ∼200 fungal genomes for putative homologs of PRC1 and PRC2 components (Table 1). Budding yeast, *Saccharomyces cerevisiae*, and fission yeast, *Schizosaccharomyces pombe*, have been intensely studied but no H3K27 methylation has been detected. This is consistent with the absence of genes for known PRC2 or PRC1 complex components in their genomes [21]. Several other model fungi, e.g. the human pathogen *Aspergillus fumigatus*, the non-pathogenic *Aspergillus nidulans* and the widely used plant pathogen *Ustilago maydis* also lack genes for known PRC components. The human pathogens *Cryptococcus neoformans* and *Cryptococcus gattii* have homologues for KMT6 and EED with other PRC components not discernable by even low-stringency BLAST searches [21]. In contrast, many filamentous ascomycetes, such as the model organism *N. crassa* [17] and the widely studied genus *Fusarium* contain a full complement of PRC2 components, with one homologue each for EZH, EED, SUZ12 and the ubiquitous Nurf-55/RbAP46/48. All fungal genomes we have investigated, however, lack Pc or other PRC1 subunits, and the components of known PRC2-targeting complexes (Table 1), suggesting that gene repression by PRC2 is mediated by a mechanism that is different from that in plants or animals [21],[22],[23].

**Table 1:** Components of Polycomb Repressive Complexes.

Drosophila	Human	*F. graminearum*	Locus ID
**PRC2**			
enhancer of zeste, E(z)	EZH2/EZH1	FgKMT6	FGSG_15795
extra sex combs, Esc/Escl	EED3/1/2/4	FgEed	FGSG_15909
suppressor of zeste 12, Su(z)12	SUZ12	FgSuz12	FGSG_04321
Nucleosome remodeling factor Nurf55	RBAP48/46	FgCAF1-3	FGSG_16720
**PRC2-associated proteins**			
polycomb-like (Pcl)	PCL1/2/3	none	N/A
Jarid2	JARID2	FgKDM5	FGSG_16557
JING (?)	AEBP2	none	N/A
**PRC1**			
polycomb (Pc)	CBX4/2/6/7/8	none	N/A
polyhomeotic (Ph)	PH1/2/3	none	N/A
posterior sex combs (Psc)	BMI1/PCGF2	none	N/A
Sex combs extra (RING)	RING1B/A	none	N/A
**PRC-targeting complexes (PhoRC)**			
Pleiohomeotic (Pho)	YY1	none	none
Sfmbt1	MBT	none	N/A

Known components from *Drosophila*, human and *F. graminearum* are shown. Apart from single homologues of all of the *bona fide* PRC2 complex subunits and a potential Jarid2 homologue, PRC1, PRC2-associated, or PRC2-targeting complexes found in animals are not conserved in fungi. Locus ID numbers are based on annotation by MIPS (http://mips.helmholtz-muenchen.de/genre/proj/FGDB/).

doi:10.1371/journal.pgen.1003916.t001

Here we focus on *F. graminearum* KMT6, the homologue of *Drosophila* E(z) and human EZH2 [22],[23], which has been identified in *N. crassa* as SET-7 [17]. Both fungal KMT6-type histone methyltransferases (HMTs) are significantly longer than the metazoan proteins (Fig. S1), and outside of the recognizable pre-SET (or CXC) and SET domains essential for HMT activity [24] there is little similarity to the metazoan proteins. Esc/EED-, Su(z)12/SUZ12- and RNA-interaction motifs found in metazoan KMT6 proteins [23] are not recognizable by sequence comparisons, yet long stretches between KMT6 proteins from various fungi are conserved, suggesting the presence of fungal-specific motifs (data not shown). The KMT6 CXC domain is characterized by cysteine repeats (four Cys-X-Cys motifs interrupted by variable length spacers and single C residues), similar to the canonical CXC motif [25], which is important for substrate recognition and enzymatic activity [24]. All four proteins have similar CXC domains (Fig. S1) when compared to other HMTs [24], suggesting that their catalytic motifs are more closely related than to HMTs with different substrates, e.g. KMT1, an H3K9-specific KMT. Important conserved stretches common to all *bona fide* HMTs are present in both KMT6 and *Neurospora* SET-7 (Fig. S1), including the invariable tyrosine that is involved in catalysis [24]. The residues preceding this tyrosine (GEELFF) are more conserved within KMT6 proteins than in HMTs in general, again suggesting that KMT6 and SET-7 are most similar to E(z) and EZH2.

### Chip-Seq Identifies Chromosomal Regions With Opposing Histone Marks

**Figure 1:**
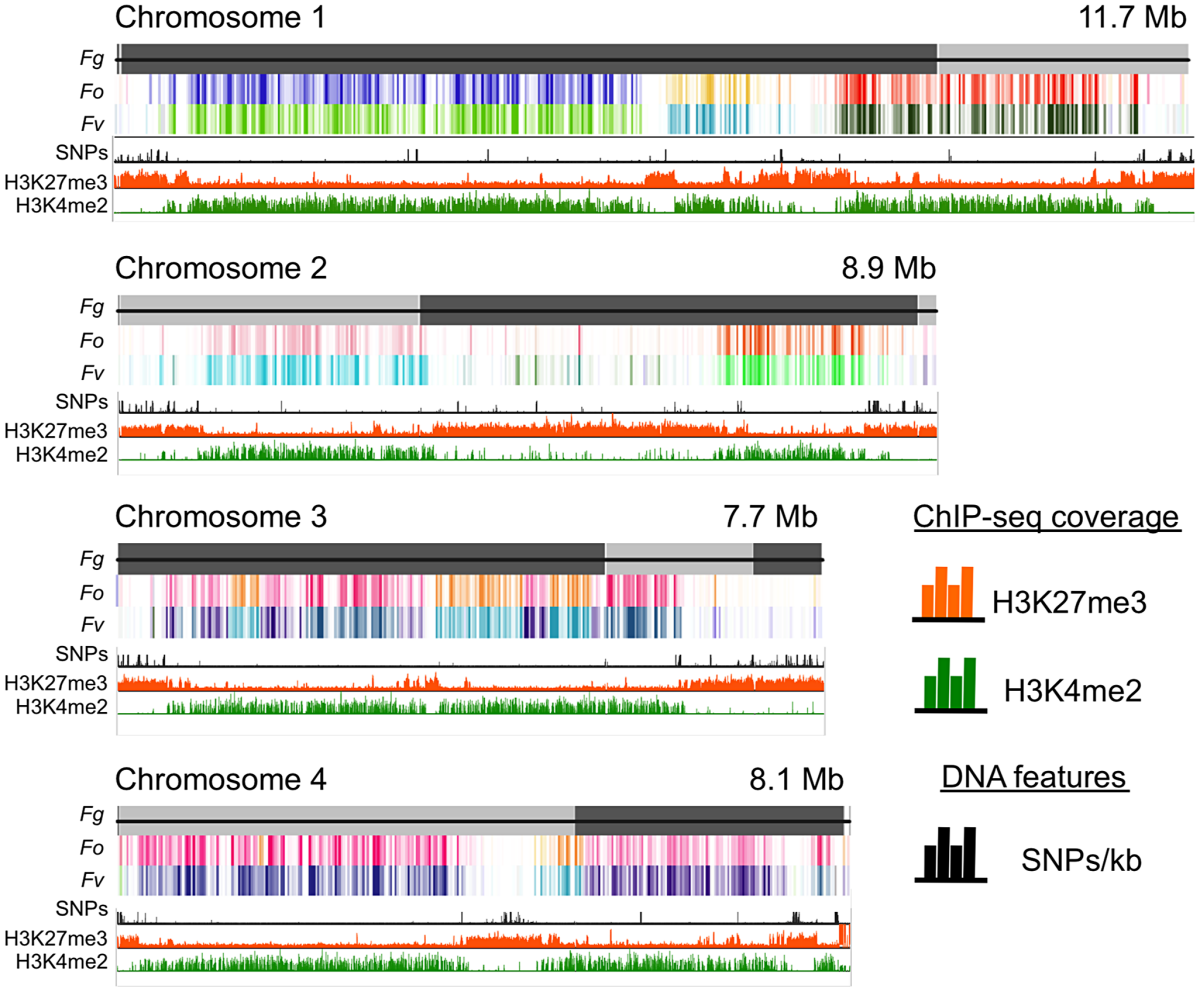
Histone marks distinguish syntenic and non-syntenic regions in Fusarium species. Synteny maps were generated at http://www.broadinstitute.org/annotation/genome/fusarium_group/Chromomap.html for *F. graminearum* PH-1 (*Fg*), *F. verticilliodes* 7600 (*Fv*), and *F. oxysporum* 4287 (*Fo*). *Fg* supercontigs are shown as alternating shades of gray on top (reference chromosomes). Regions of synteny between *Fg*, and each chromosome of *Fv* and *Fo* are shown in different colors where a particular color represents a specific chromosome; white indicates absence of synteny. To show regions of increased recombination, SNPs/kb observed in *Fg* strain 00-676 compared to the reference strain PH-1 are shown as black hatches. ChIP-seq results (reads/kb) are shown for H3K27me3 (orange) and H3K4me2 (green).

To understand genome organization of *F. graminearum*, we performed ChIP-seq with antibodies against histone modifications known to be associated with active (H3K4me2) or silent (H3K9me3 and H3K27me3) chromatin. H3K4me2 and H3K27me3 were found in large, mutually exclusive, gene-rich blocks of the genome (Fig. 1). About one third of the *F. graminearum* genome is associated with H3K27me3 when the fungus is grown in minimal medium with low nitrogen; global H3K27me3 enrichment is slightly reduced in high nitrogen medium. More than half (58%) of chromosome 2 is covered by this silencing mark (Fig. 1).

Comparative genome studies suggested that the four chromosomes of *F. graminearum* are the result of chromosome fusion events, as the most closely related *Fusarium* species have between 11 and 15 chromosomes each, and SNP maps between two different strains suggested recombination patterns that mark some internal regions of *F. graminearum* as ancestral subtelomeres [26],[27]. We thus constructed synteny maps between *F. graminearum*, *F. oxysporum*, and *F. verticillioides* and compared them to histone modification maps (Fig. 1). H3K4me2 (green track) is found in well-conserved regions with high synteny between the three species. In contrast, H3K27me3 (orange track) is found in non-syntenic blocks unique to *F. graminearum*, predominantly in subtelomeric regions. In agreement with the chromosome fusion hypothesis, we found extended internal blocks of H3K27me3 on each chromosome that may constitute ancestral subtelomeric regions. H3K27me3 colocalizes with regions of high SNP density between the reference genome strain (PH-1) and a second wild-collected strain that we re-sequenced, 00-676 [28] (Fig. 1, black histogram). In complementary experiments we observed similar patterns for the distribution of H3K27me3 in other *Fusarium* species, e.g. *F. verticillioides*, *F. asiaticum* and *F. fujikuroi* (L.R. Connolly, L. Studt, K.M. Smith, B. Tudzysnki, S.-H. Yun and M. Freitag, unpublished data). Subtelomeric regions in filamentous fungi are enriched for lineage- or species-specific genes, for example secondary metabolite gene clusters, as well as genes for secreted pathogenicity factors and detoxifying enzymes [29],[30]. Our observations led us to ask if genes enriched for H3K27me3 are repressed, and if these genes become active when H3K27me3 is removed by mutation of KMT6.

### Deletion Of *Kmt6*, A Gene Encoding A Set-Domain Histone Methyltransferase, Eliminates H3k27 Methylation

We identified the *F. graminearum kmt6* gene (FGSG_15795.3) based on BLAST searches with *Drosophila* E(z). We used targeted gene replacement to disrupt *kmt6* (Fig. S2A). PCR and Southern analyses confirmed replacement of *kmt6* in colonies that were exceptionally orange in pigmentation, suffered aberrant germination patterns and stunted growth. Southern blots showed absence of the *kmt6* gene and replacement with *neo* ^+^ (confers G418 resistance) in the mutant transformant (Fig. S2A). A *kmt6* mutant (FMF248) with perfect *neo^+^* integration was chosen for further studies.

**Figure 2:**
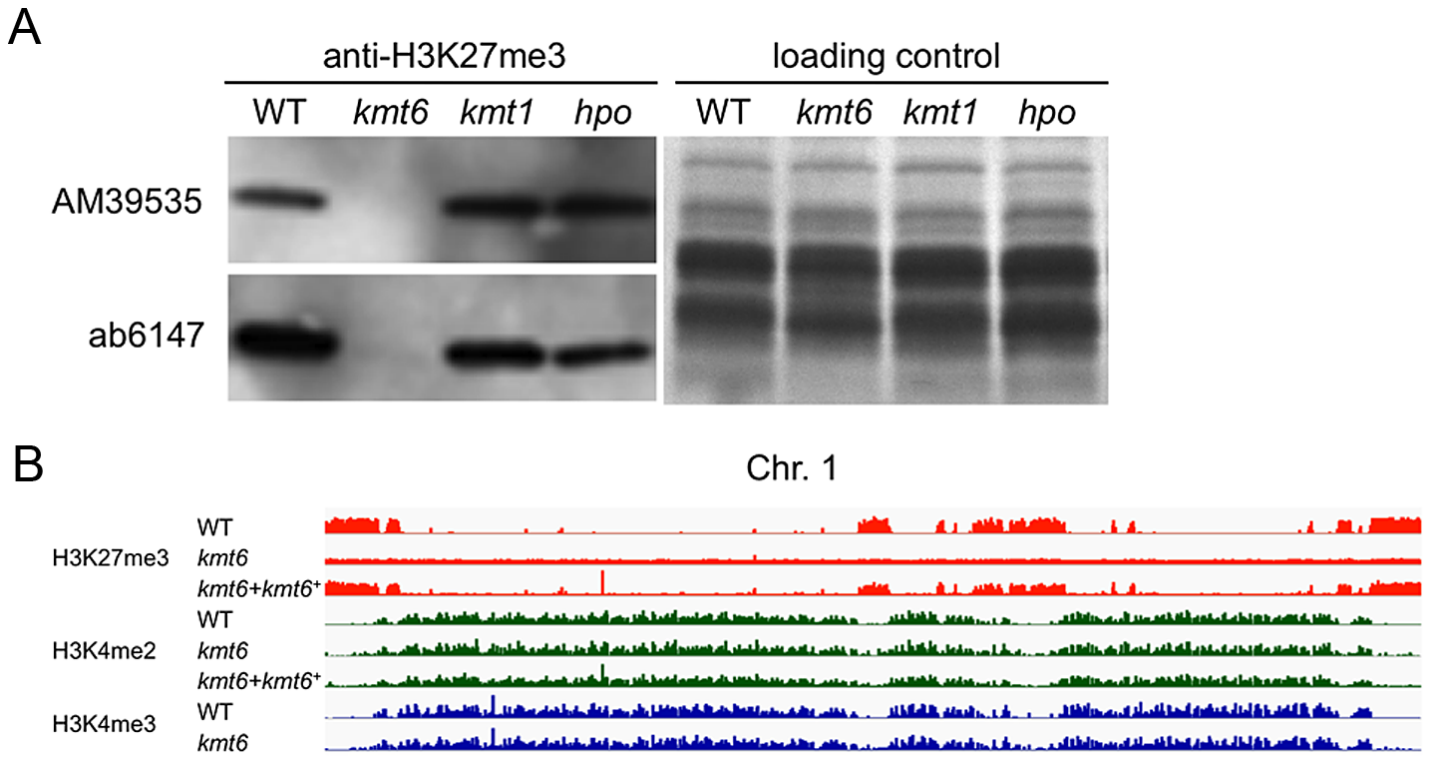
The *kmt6* mutant lacks H3K27me3. A. Western analyses with acid-extracted histones from WT or *kmt6*, *kmt1* and *hpo* mutants show absence of H3K27me3 in *kmt6* but unchanged levels in *kmt1*, a mutant deficient in the H3K9 methyltransferase homologue of Su(var)3-9, Clr4 and DIM-5, and in *hpo*, a mutant in which the homologue of Heterochromatin Protein 1 (HP1) was deleted (L.R. Connolly and M. Freitag, in preparation). Two different antibodies (AM39535 and ab6147) were used. B. H3K27 trimethylation occurs in large blocks. H3K27me3 (orange) is mutually exclusive of H3K4me2 (green) and -me3 (blue), which are found in the same regions of chromosome 1 (see Fig. S3 for images of Chr. 2 to 4). Specific enrichment of H3K27me3 is lost in the *kmt6* mutant but regained in a complemented strain (*kmt6^+^*).

To test if H3K27 methylation was altered in the *kmt6* mutant, we purified histones and carried out western analyses with antibodies against methylated histones. H3K27me3 was present in WT but completely absent from the *kmt6* mutant (Fig. 2A). We tested several different antibodies raised against H3K27me3 peptides (Fig. 2A, Fig. S2B). All showed absence of H3K27me3 in *kmt6*. Levels of H3K4me2 and another activating mark, H3K36me3, are equivalent in WT and *kmt6* (Fig. S2B), suggesting that lack of H3K27me3 does not result in an overall increase in H3K4 or H3K36 methylation. H3K9me3, while present, proved difficult to detect in *F. graminearum* by western blot (Fig. S2B), matching our expectations from ChIP-seq (data not shown). Levels of H3K27me3 were not altered in strains in which H3K9me3 was abolished by deletion of the single *Fusarium* Su(var3-9) homologue (*kmt1*) or in which Heterochromatin Protein-1 (HP1) was deleted (*hpo*) (Fig. 2A). We conclude that KMT6 has specificity for H3K27, and that KMT6 is the sole or predominant H3K27 methyltransferase in *F. graminearum*.

We repeated ChIP-seq of H3K4me2 and H3K27me3, as well as H3K4me3 and H3K36me3, in WT, *kmt6* and the complemented strain under nitrogen limiting and nitrogen abundant conditions (Fig. 2B, Table S1). We used different nitrogen levels as one environmental factor that is known to affect gene regulation in many fungi. In the *kmt6* mutant, H3K27me3 enrichment was completely lost, and only background genomic sequence was obtained by ChIP-seq (Fig. 2B, Fig. S3). After re-introduction of a wildtype *kmt6* allele, H3K27me3 was restored to levels almost indistinguishable from WT. Our results confirm that H3K27me3 is generated by KMT6, and that restoring gene function restores H3K27me3 enrichment in all regions by *de novo* mechanisms that may be similar to those in animals or plants.

Certain blocks previously enriched with H3K27me3 showed acquisition of H3K4me2/3 in *kmt6* mutants. No obvious differences in enrichment were observed between high and low nitrogen conditions when viewed at the whole chromosome level. Similarly, discrete H3K36me3 enrichment in genic regions was not resolvable at the whole chromosome scale. H3K4me2 and H3K4me3 were found in overlapping regions, mutually exclusive of H3K27me3.

### The *Kmt6* Mutant Shows Developmental Defects

**Figure 3:**
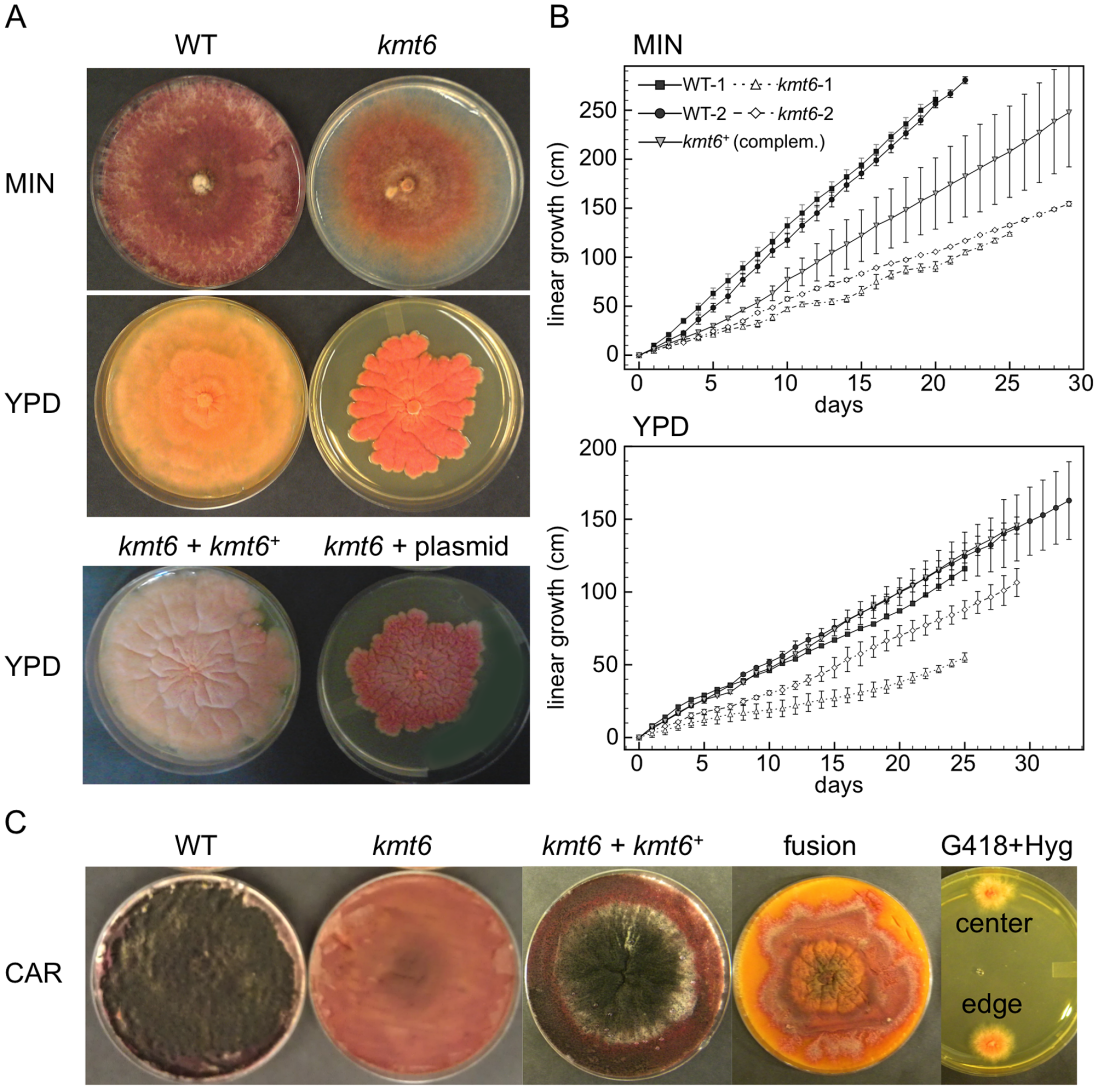
The *kmt6* mutant displays altered growth and development. A. WT and *kmt6* strains were grown on minimal (MIN) or YPD medium. The mutant shows sparse growth on MIN and dense, slow-growing and intensely orange mycelium on YPD. The lower panel shows YPD plates with a complemented strain (*kmt6*+*kmt6^+^*) and a plasmid control (*kmt6*+plasmid). B. WT and *kmt6* were grown through race tubes on MIN or YPD to measure linear growth for 25 to 33 days. Results are the average of three replicates, and bars indicate standard error. C. Sexual development was induced on carrot agar (CAR) plates. Black patches in the WT plate indicate numerous fully developed perithecia, while no perithecia formed in *kmt6* plates. The complemented strain (*kmt6*+*kmt6^+^*) is able to produce perithecia and ascospores at similar levels as WT. Protoplast fusions to complement *kmt6 neo^+^* strains with WT *hph^+^* nuclei do not restore formation of perithecia. These heterokaryons did not break down, as colonies resistant to both G418 and Hyg were isolated from different areas of the CAR plate and grew on YPD+G418+Hyg (center and edge).

All *kmt6* mutants obtained are sterile, have morphological defects, and are altered in pigment production. We observed slower linear growth and morphological changes in *kmt6* compared to WT on both minimal (MIN) and rich (YPD) media (Fig. 3A–B). Plates are covered by WT after a few days, so we used Ryan (“race”) tubes [31] to carry out long-term growth experiments. We measured linear extension of WT and *kmt6* for one month (Fig. 3B). On both plates and in race tubes, linear growth on minimal medium was faster than on the richer YPD medium, but colonies on YPD grew more densely. Overall, WT growth was more than two-fold faster than *kmt6* growth. We compared growth of *kmt6* and WT in a wounded tomato assay [32] and found that *kmt6* was unable to colonize fruit (Fig. S4), suggesting that the mutant has reduced pathogenicity.

To show that *kmt6* was responsible for the defects described, we complemented a mutant strain (FMF248) with a wildtype allele of *kmt6* flanked by the *hph^+^* gene (confers hygromycin resistance; Fig. S2A). The complemented strain (FMF282) retains the *neo* marker at the endogenous *kmt6* locus, but has an insertion of *kmt6* and *hph* at an ectopic locus. The intensity of *kmt6* probing and multiple hybridizing bands in the complemented strain suggest multiple tandem insertions of the wildtype *kmt6* gene. The complemented strain showed intermediate growth rates, faster than the mutant but not fully restored to WT growth levels, and almost normal pigmentation on minimal medium (Fig. 3 A–B). On rich medium (YPD), WT grew roughly two fold faster than *kmt6*; on this medium the complemented strain grew as well as WT (Fig. 3B).

*Fusarium graminearum* is a homothallic, or self-fertile, fungus. When placed on carrot agar (CAR), WT strains undergo sexual development to generate dark pigmented fruiting bodies, “perithecia” (Fig. 3C, CAR). Depending on environmental conditions (temperature, humidity), a selfing takes 10 to 14 days. After this time, ripe ascospores are shot or ooze from perithecia in cirrhi [33],[34]. We found that *kmt6* is completely infertile and does not undergo even the earliest stages of sexual development. The complemented strain initiated normal development, though production of ascospores took about twice as long as for WT strains (Fig. 3C).

We attempted to force heterokaryons between *kmt6::neo^+^* and *hph^+^* strains of the same lineage. When co-inoculated, heterokaryons never formed on selective medium, suggesting anastomosis defects; only the *hph^+^* sectors were able to generate perithecia with viable ascospores. We carried out protoplast fusions to complement the *kmt6* deletion by formation of [*kmt6*+*kmt6^+^*] heterokaryons (Fig. 3C). No sexual development was observed even after extended periods of incubation, unlike for wildtype or complemented strains, which look similar to regular selfings but take 2–3 days longer to mature. We were able to isolate double-resistant G418^+^ and Hyg^+^ colonies from both the edge and center of the [*kmt6*+*kmt6^+^*] colonies on carrot agar, indicating that the heterokaryon had not broken down (Fig. 3C). We conclude that the sexual differentiation defect can be complemented by transformation but surprisingly not by fusion with mycelia that should be competent for sexual development. These experiments suggest existence of dominant factors produced by *kmt6* nuclei that may inhibit H3K27me3 regulation in *kmt6^+^* nuclei or act as dominant factors inhibiting sexual development. Action of these factors cannot be easily overcome by hyphal fusion and further studies to unravel this gene regulatory developmental switch are underway.

### Histone Modifications Are Differentially Enriched Across Genes

**Figure 4:**
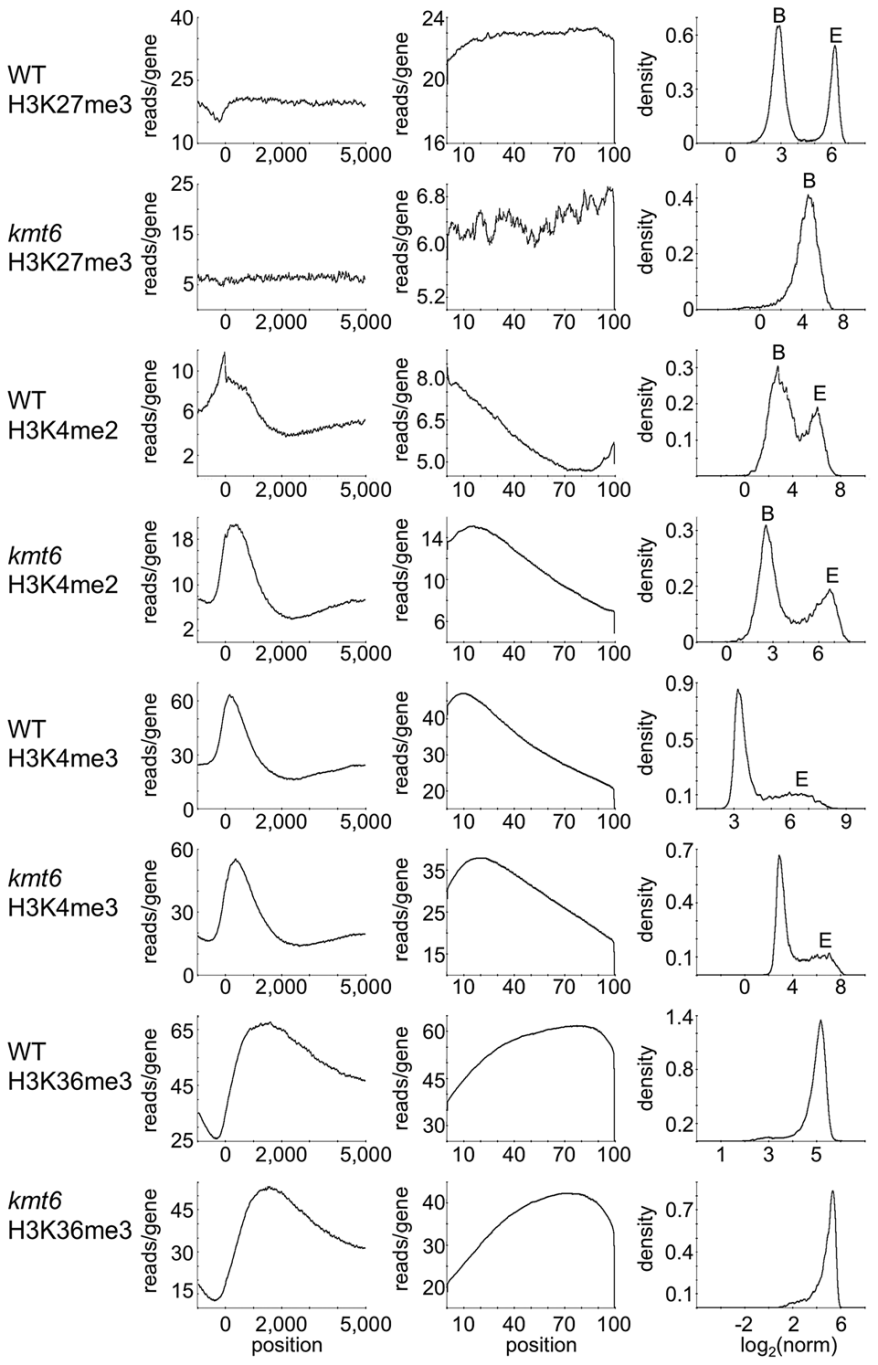
Distribution of histone modifications across the complete set of *F. graminearum* genes. The left panel for each modification shows read counts (reads/gene) across all genes that were aligned to make the known or presumed transcriptional start site (TSS) position “0”. The middle panel shows read counts across all genes (reads/gene) normalized by gene length (x-axis indicates percent of gene length). The right panel shows the distribution of normalized read counts (NLCS; density) per gene. For most modifications two peaks are observed, background levels of reads (“B”) and enrichment (“E”). Results are for low nitrogen experiments, but indistinguishable results were obtained with samples grown on high nitrogen medium.

To distinguish genes that are enriched for a particular histone modification from those with background levels of ChIP-seq reads we used EpiChIP [35], which calculates values for “normalized locus chromatin state” (NLCS) and false discovery rates for each gene. The NLCS is the area under each ChIP-seq peak, in a specified window, normalized for the length of the window and the sequencing depth. For our analysis we used genes from the current Broad Institute annotation (http://www.broadinstitute.org/annotation/genome/fusarium_group/MultiHome.html) as the window, without addition of upstream or downstream sequences. For most histone modifications we found two peaks in the NLCS distribution (Fig. 4, right panels), one for background signals (B) and one for enrichment (E). In WT, H3K27me3 enrichment extended across gene bodies but was absent near the TSS, and genes with background levels (log_2_∼3) are clearly distinguishable from genes with enrichment (log_2_∼6). In *kmt6*, only background signal remained for H3K27me3, resulting in a single peak in the NLCS distribution. As expected, H3K4me2 and -me3 enrichment were most pronounced near the 5′ end of genes, while H3K36me3 was found more enriched near the 3′ end of genes (Fig. 4). A single peak is observed in the distribution of H3K36me3, but unlike H3K27me3 in *kmt6*, the single peak in H3K36me3 NLCS distribution represents enrichment. The *kmt6* mutant revealed similar patterns of enrichment for H3K4 and K36 methylation across genes when compared to WT.

### Loss Of H3k27me3 Releases Transcriptional Inhibition

**Figure 5:**
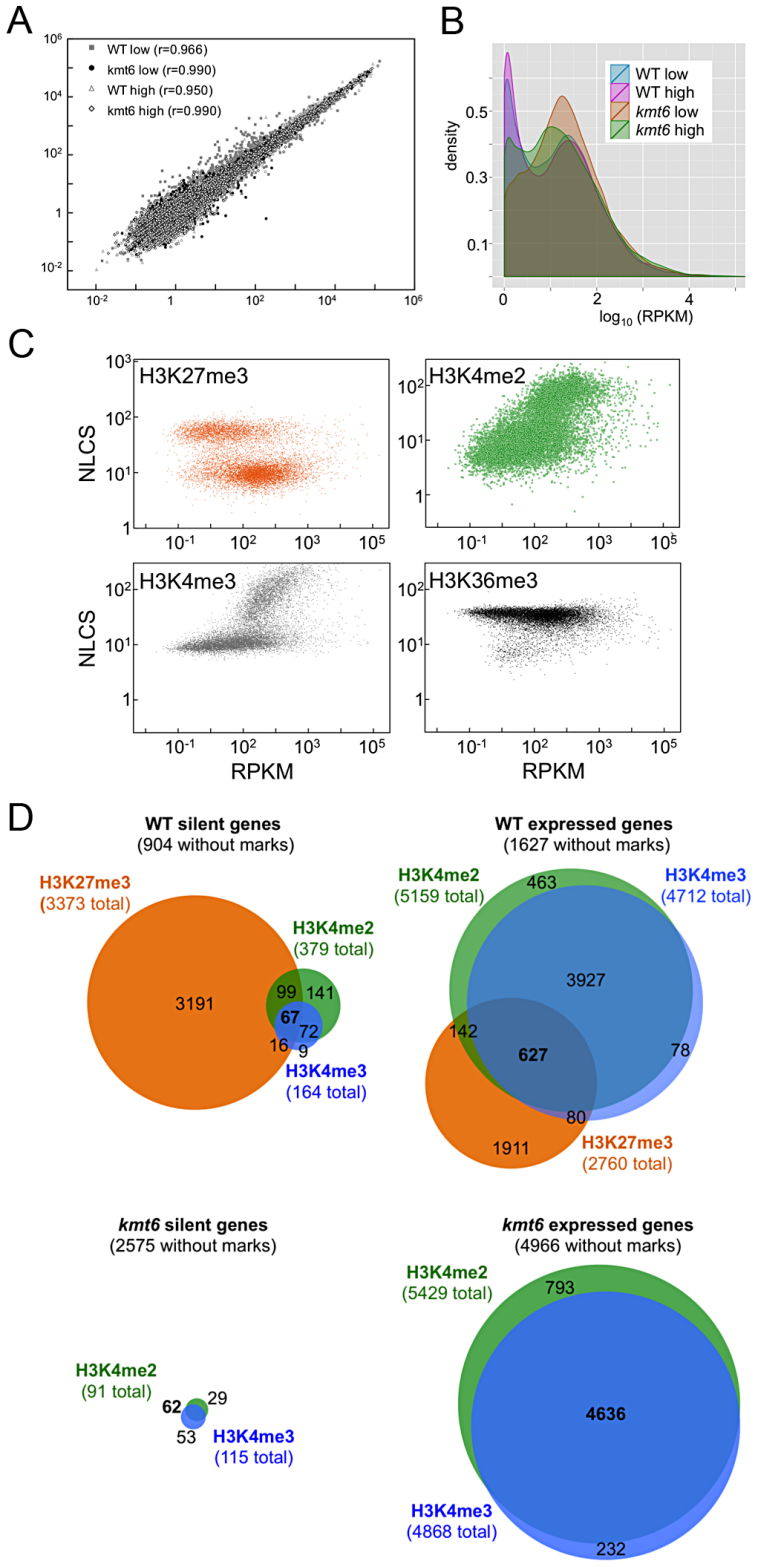
Global transcriptional analysis and correlation with histone modifications. A. Biological replicates are highly correlated. The FPKM for biological replicate 1 is plotted against biological replicate 2 for each gene, for all four conditions, demonstrating strong correlation between replicate experiments (r is the correlation coefficient). B. Pairwise comparisons of RPKMs for each gene, WT *vs. kmt6* and low *vs.* high nitrogen for each strain shows global changes in gene expression. While there are some changes in expression based on nitrogen availability, more drastic changes are observed between WT and *kmt6* (orange and green area). C. Histone modifications are correlated to expression state. For each gene, expression (RPKM) is plotted *vs.* histone enrichment (NLCS) for H3K27me3 (orange), H3K4me2 (green), H3K4me3 (gray) and H3K36me3 (black) for WT grown in low nitrogen. For each modification, except for H3K36me3, two clusters of points can be seen on the y-axis, correlating to the background and enriched gene groups from the distribution curves (see Fig. 5). D. Histone modifications associated with silent and expressed genes in low nitrogen conditions. Genes were classified as silent (≤3 RPKM) or expressed (>3 RPKM) and enrichment of H3K27me3, H3K4me2, and H3K4me3 determined by EpiChIP NLCS values. Venn diagrams were drawn to show histone enrichment for each silent or expressed gene in WT (A–B) or *kmt6* (C–D).

We performed RNA-seq on WT and *kmt6* strains in high and low nitrogen conditions to investigate whether gene expression correlates with histone modifications in the expected manner. We used Tophat to map reads obtained by RNA-seq, and cufflinks to calculate reads per kilobase of transcript per million mapped reads (RPKM), a value representative of gene expression and normalized for both transcript length and sequencing depth [36]. For each condition we plotted the RPKM of each gene from each of two biological replicates (Fig. 5A). For most genes the replicates produced similar RPKM values, and all points fall near a line with a slope of 1. Not unexpectedly, most of the variation was observed in genes with low expression. Comparing expression of genes from WT or *kmt6* at low compared to high nitrogen (Fig. 5B) showed that a relatively small percentage of genes has altered expression in response to nitrogen levels. Overall we observed a trend toward decreased gene expression in low nitrogen, shown by the smooth fit regression line (Fig. S5). The *kmt6* mutation caused a larger change in global gene expression than changing nitrogen availability, a well-studied environmental factor affecting expression of known metabolites [37]. The overall trend was towards increased gene expression in *kmt6*. The distribution of RPKM values for all genes in each condition revealed that high nitrogen caused repression of only 5–10% of all genes in both strains, while the *kmt6* mutation released repression of 15–30% of all genes; many of these were repressed by high nitrogen levels (Fig. S5).

To address if histone modifications are truly predictive of gene expression in *F. graminearum*, we show the range of RPKM values plotted against enrichment of histone modifications, expressed as the normalized NLCS values from EpiChIP (Fig. 5C). Overall, H3K27me3 enriched genes had low RPKM values; most genes enriched for H3K27me3 (NLCS>16) had RPKM values <10, indicating very low expression. There are several genes, however, with high H3K27me3 enrichment and RPKM>100, suggesting gene expression in the presence of a usually silencing histone modification. As one would expect, genes with high values of enrichment for H3K4me2/3 also tended to have higher RPKM values, and for both H3K4me2 and H3K4me3 genes with no enrichment tended to have low expression levels, suggesting a stronger correlation between enrichment with H3K4me2/3 and expression than presence of H3K27me3 and silencing. H3K4me2 was found in far more genes than H3K4me3. H3K36me3 was found in nearly all genes, regardless of expression level.

We classified genes grown in low nitrogen as expressed or silent in both WT and *kmt6* strains based on the distribution of RPKM values (Fig. 5C). When comparing WT to mutant, we found that in WT 8,855 (or 66% of all annotated 13,354) genes were expressed (Fig. 5D). Of these, 1,627 genes were not associated with any of the histone modifications investigated. More than 30% of all expressed genes (2,760 of 8,855) were significantly enriched for the silencing H3K27me3 mark, though many of these genes showed H3K27me3 enrichment just above background levels. The 4,449 silent genes (33% of 13,354 annotated genes) in WT were largely associated with H3K27me3 (76% or 3,373 of 4,499), but almost 200 silent genes also had some significant H3K4me2/3 enrichment. When H3K27me3 is lost by *kmt6* mutation, the number of expressed genes jumps to 10,635 (Fig. 5D); this number does not include genes that are expressed in WT yet are more highly expressed in *kmt6*. Only half of these genes are newly enriched for H3K4me2/3. The other half has none of the investigated modifications. Overall we found that about 14% of the genome is derepressed by absence of H3K27me3; many additional genes are overexpressed in *kmt6* compared to WT.

### Kmt6 Is A Repressor Of Secondary, Not Primary, Metabolism

**Figure 6:**
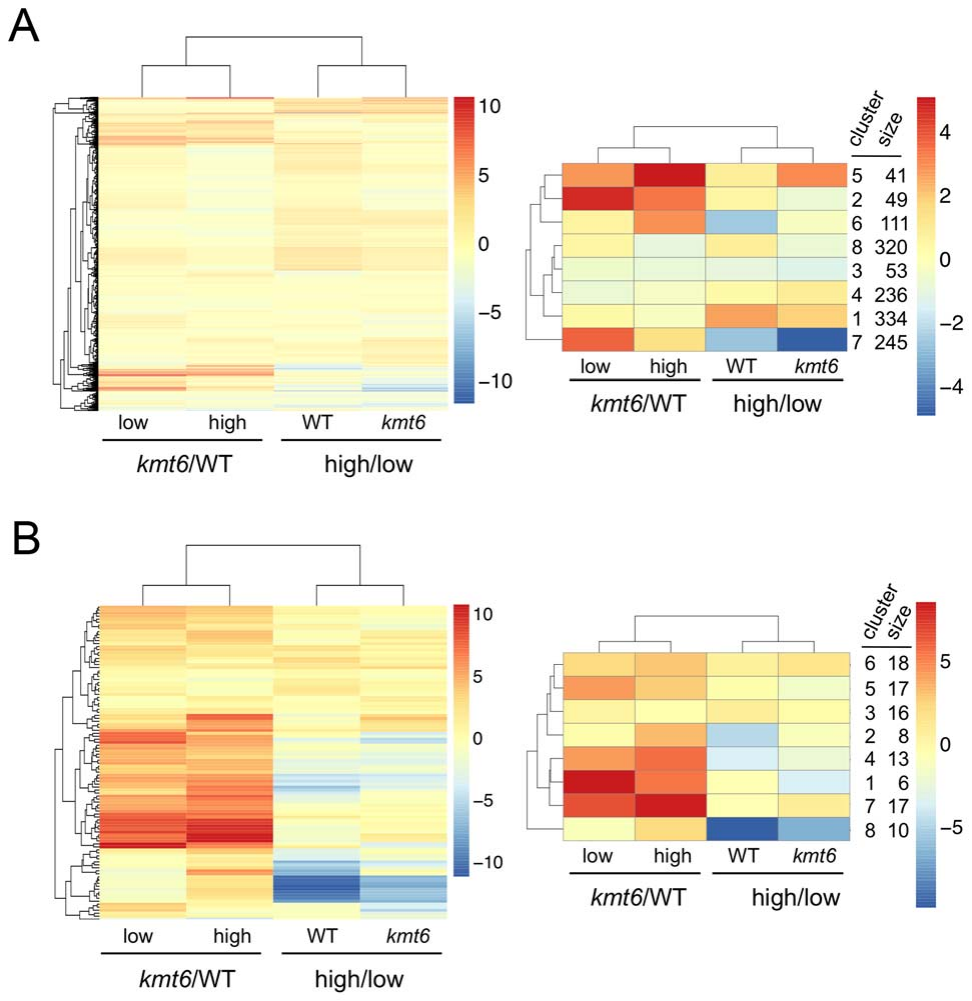
Heatmaps for individual genes in primary (A) and secondary (B) metabolism (left panels) and clusters of genes around eight centers (right panels). Few primary metabolic genes are affected by *kmt6* mutation compared to the majority of secondary metabolic genes that have increased expression in *kmt6*. Values for the log_2_ of the ratio of RPKMs for *kmt6*/WT or high/low nitrogen conditions for each strain are color-coded and the scale shown on the right. For example, in the left image of (A) red represents log_2_ (*kmt6*/WT) of 10, which means the *kmt6* RPKM is 1024-fold higher than the WT RPKM. Primary metabolic genes in clusters 2 and 5, upregulated in *kmt6*, and cluster 7, downregulated in high nitrogen, are listed in Table S2 with their putative functions. Secondary metabolic genes in clusters 1, 4, and 7, upregulated in *kmt6*, and cluster 8, downregulated in high nitrogen, are listed in Table S2 with their putative functions.

We immediately realized that regions of KMT6-dependent repression are home to secondary metabolite (SM) gene clusters, and thus generated heatmaps of expression changes for all primary and secondary metabolite genes to visualize the effect of high compared to low nitrogen and *kmt6* mutation on expression of these genes. Growth in low and high nitrogen was compared because nitrogen is a known regulator of many SM gene clusters [37]. Primary metabolite (PM) genes were largely unaffected by either mutation of *kmt6* or growth in high nitrogen (Fig. 6A). Specific sets of genes, summarized in the clustered heatmap with 8 k-means (Fig. 6A, right panel) stand out as being repressed in high nitrogen (245 genes in cluster 7), or induced by *kmt6* mutation (41 genes in cluster 5 and 49 genes in cluster 2). The genes repressed in high nitrogen include six out of 17 genes in the gluconeogenesis I pathway, and several genes for carbohydrate metabolism including glycolytic enzymes (Table S2). The 90 genes induced in *kmt6* are enriched for genes involved in carbohydrate binding and degradation, peptidases, and cell signaling components. Five of the 90 genes, although classified as primary metabolic genes, are part of SM clusters (*carB*, *fus1*, *tri5*, a kinase belonging to the *zon* pathway, and FGSG_10615).

In contrast to PM genes, the complete set of SM genes (Fig. 6B) was overall more derepressed in *kmt6* (34% of SM genes compared to 6% of PM genes), but a smaller fraction was repressed in high nitrogen (10% of SM genes compared to 18% of PM genes). The ten genes repressed in high nitrogen (Table S2) include most of the aurofusarin cluster (*aurO*, *aur1*, *aurC*, *aurJ*, *aurF*, *gip1* and *aurS*), plus an ammonium permease from the carotenoid cluster, as well as *pks1* and a multidrug resistance protein, both from SM cluster FG3_38, which generates an unknown product [27]. Since our nitrogen source was ammonium nitrate, it is not surprising that an ammonium permease was downregulated. The 36 genes derepressed in *kmt6* (Table S2) include *carO* and *carX* from the carotenoid cluster, nine genes from the fusarin C cluster (*fus1*, *fus2*, *fus3*, *fus5*, *fus6*, *fus7*, *fus8*, and two other unnamed genes), five genes from cluster FG3_20 and six genes from FG3_40, which both generate unknown products [27]. These 36 genes are the most derepressed SM genes in *kmt6* (log_2_ fold change >4, or more than a 16-fold induction). Many other genes are derepressed to a smaller but still significant degree.

**Figure 7:**
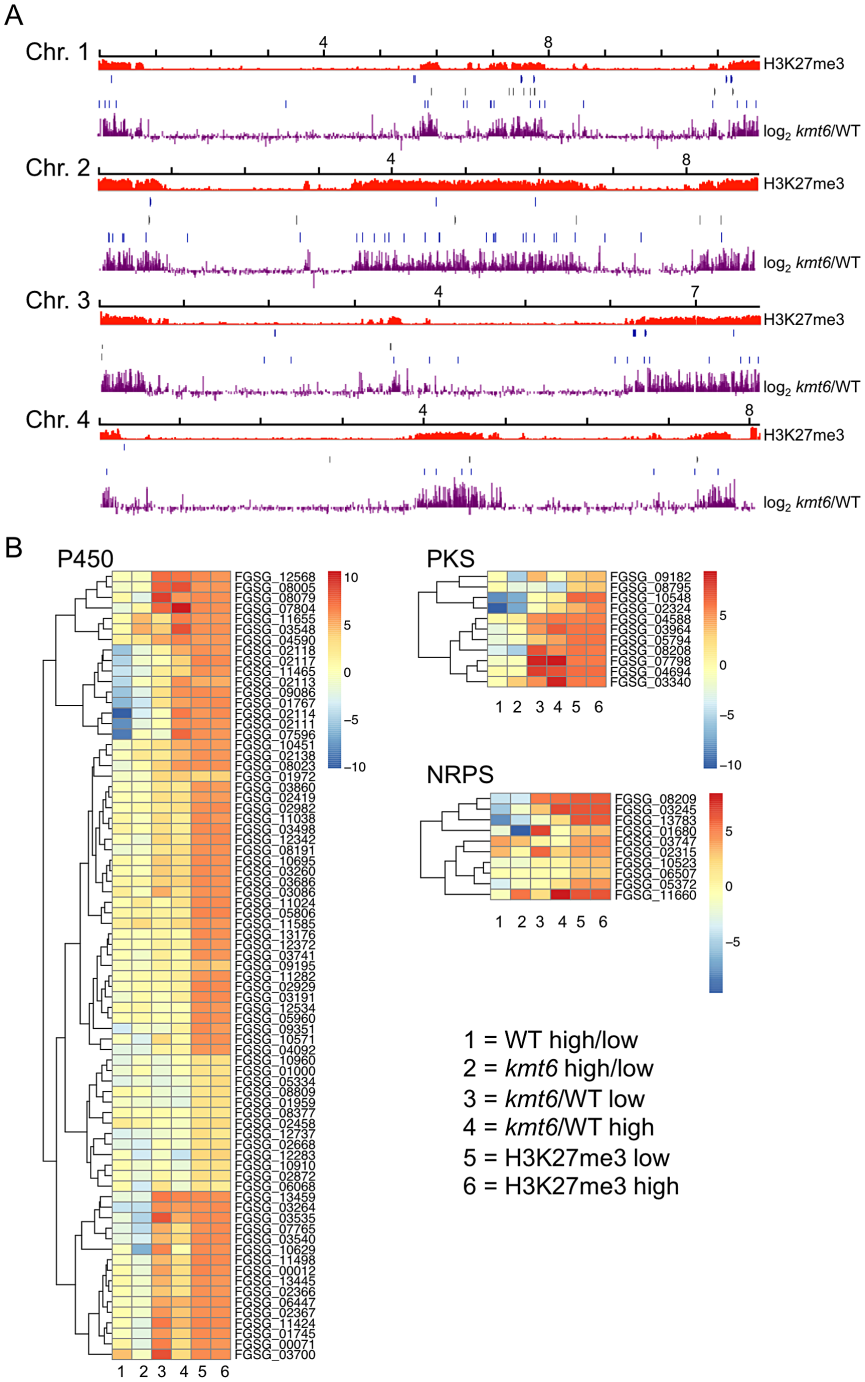
Genes involved in secondary metabolism are found in regions of H3K27me3, but not all are induced by mutation of *kmt6*. A. NRPS (blue, top), PKS (gray, center) and Cytochrome P450 (light blue, bottom) genes were mapped to the four chromosomes, in relation to H3K27me3 (low nitrogen conditions). Almost all genes are in regions covered by H3K27me3. . Changes in transcription for low nitrogen conditions are shown in purple as log_2_ value of the RPKM ratio of *kmt6 vs.* WT. B. Heatmaps showing expression changes and H3K27me3 enrichment of three classes of SM genes, cytochrome P450s, PKSs, and NRPSs. Legends to heatmaps as described in Fig. 8. H3K27me3 enrichment is shown as log_2_ value of the NLCS.

To show that SM genes are found most often in KMT6-repressed regions we mapped genome-wide changes in gene expression (log_2_ *kmt6*/WT), distribution of H3K27me3, and genes for cytochrome P450 enzymes and gene clusters containing polyketide synthases (PKS) or non-ribosomal peptide synthases (NRPS) onto *F. graminearum* chromosomes (Fig. 7A). We also generated heatmaps of expression data for these groups of genes (Fig. 7B). Families of cytochrome P450s and PKSs are proposed to have evolved by gene duplication and divergence [38],[39]. Most cytochrome P450, PKS, and NRPS genes were enriched for H3K27me3 (Table 2). Several, but not all, cytochrome P450s were derepressed in *kmt6*, notably *tri4* (FGSG_03535) and *tri11* (FGSG_03540) involved in deoxynivalenol (DON) synthesis, and *fus8* (FGSG_07804) in the fusarin C pathway. Other cytochrome P450 genes were repressed in high nitrogen, and generally derepressed in *kmt6* to a much greater degree than when comparing high to low nitrogen conditions. These include a block of contiguous genes on chromosome 1, FGSG_02111, FGSG_02113, FGSG_02114, FGSG_02117, and FGSG_02118. The products of these genes are unknown, but the neighboring gene, FGSG_02115, encodes a TRI7 (toxin biosynthesis protein) homolog and FGSG_02116 encodes an NAD-dependent epimerase or dehydratase, suggesting the existence of a novel SM cluster.

**Table 2:** Summary of expression data for 45 secondary metabolite clusters of *F. graminearum*.

				H3K27me3 enrichment	Nitrogen repression	Expression	
FGSG#	Class	Gene	Product			in *kmt6*	in WT
FGSG_11026	NRPS 1		malonichrom	yes	no	no	no
FGSG_05372	NRPS 2	*nps2*	ferricrocin	yes	yes	yes	in low N
FGSG_10523	NRPS 3			no	no	no	no
FGSG_02315	NRPS 4	*nps4*		yes	yes	yes	in high N
FGSG_13878	NRPS 5			yes	no	no	no
FGSG_03747	NRPS 6	*nps6*	fusarinine	yes	yes	in high N	in high N
FGSG_08209	NRPS 7	*nps7*		yes	yes	yes	in low N
FGSG_11659	NRPS 8	*nps8*		yes	yes	in high N	no
FGSG_10990	NRPS 9			yes	no	yes	no
FGSG_06507	NRPS 10	*nps10*		no	no	yes	constitutive
FGSG_03245	NRPS 11	*nps11*		yes	yes	yes	in low N
FGSG_11294	NRPS 12			yes	yes	in low N	in low N
FGSG_13153	NRPS 13			no	no	no	constitutive
FGSG_11395	NRPS 14			yes	no	yes	no
FGSG_02394	NRPS 15			yes	no	no	no
FGSG_01680	NRPS 16			no	no	in high N	no
FGSG_10702	NRPS 17			yes	no	no	no
FGSG_13783	NRPS 18			yes	yes	in low N	in low N
FGSG_11989	NRPS 19		AM toxin	no	no	in low N	no
FGSG_04694	PKS 2	*pks2*		yes	no	yes	no
FGSG_09182	PKS 3	*pks3/pgl1*	fusarubin	no	no	in low N	no
FGSG_05794	PKS 5	*pks5*		yes	no	no	constitutive
FGSG_08795	PKS 7	*pks7*		no	yes	no	in low N
FGSG_07798	PKS 10	*pks10/fus1*	fusarin C	yes	yes	yes	in low N
FGSG_03340	PKS 13	*pks17*		yes	no	in high N	no
FGSG_10548	PKS 21	*pks1*		yes	yes	in low N	in low N
FGSG_12126	PKS 22	*pks4/zea2*	zearalenone	yes	no	yes	no
FGSG_08208	PKS 23	*pks6*		yes	yes	in low N	in low N
FGSG_10464	PKS 24	*pks9*	fusarielin	yes	yes	in low N	in low N
FGSG_01790	PKS 25	*pks11/fum1*	fumonisin	yes	yes	in low N	in low N
FGSG_02324	PKS 26	*pks12/aur1*	aurofusarin	yes	yes	in low N	in low N
FGSG_02395	PKS 27	*pks13/zea1*	zearalenone	yes	yes	no	no
FGSG_03964	PKS 28	*pks14/grs1*		yes	no	yes	no
FGSG_04588	PKS 29	*pks15/plsp1*		yes	no	yes	no
FGSG_03066	DTC1	*carA/al2*	carotenoids	yes	yes	yes	in high N
FGSG_07673	STC1			yes	yes	yes	in high N
FGSG_08181	STC5			yes	no	in low N	no
FGSG_11327	STC6		caryophylene	yes	no	no	no
FGSG_00451	STC2			no	no	no	no
FGSG_03494	STC4		acorenol	yes	yes	in high N	in high N
FGSG_10397	STC9		longiborneol	no	no	no	no
FGSG_03532		*tri8*	trichothecene	yes	no	in high N	constitutive
FGSG_05948			terpenoid	no	no	no	constitutive
FGSG_10616			terpenoid	yes	yes	in low N	in low N
FGSG_01735			terpenoid	yes	yes	yes	in low N

The 19 non-ribosomal peptide synthase (NRPS), 15 polyketide synthase (PKS) and 11 terpenoid-producing clusters were arranged into classes according to recently published nomenclature [37]. Products are known or predicted based on similarity of gene clusters with other *Fusarium* species. The last four columns summarize our results, showing enrichment with H3K27me3, repression by high nitrogen levels, and expression patterns in *kmt6* and WT for key cluster genes.

doi:10.1371/journal.pgen.1003916.t002

The effects of KMT6 on the expression of known SM clusters with NRPS, PKS, DTC and STC signature genes are summarized and contrasted to the effects of nitrogen (Table 2). Of 45 clusters, 35 are enriched with H3K27me3 in both low and high nitrogen conditions, compared to 21 of 45 that are repressed by high nitrogen levels. In *kmt6*, 32 of the 45 clusters are expressed with low (11/32), high (6/32) or either nitrogen levels (15/32). In contrast, in WT only five clusters are expressed constitutively, five are expressed in high nitrogen, 14 in low nitrogen and 21 remained silent regardless of the nitrogen level. Most of these gene clusters have unknown functions and putative compounds generated have not been defined for 29 of the 45 clusters shown. Overall, manipulation of H3K27me3 levels proved more successful for expressing these “cryptic” clusters than changes in nitrogen level.

**Figure 8:**
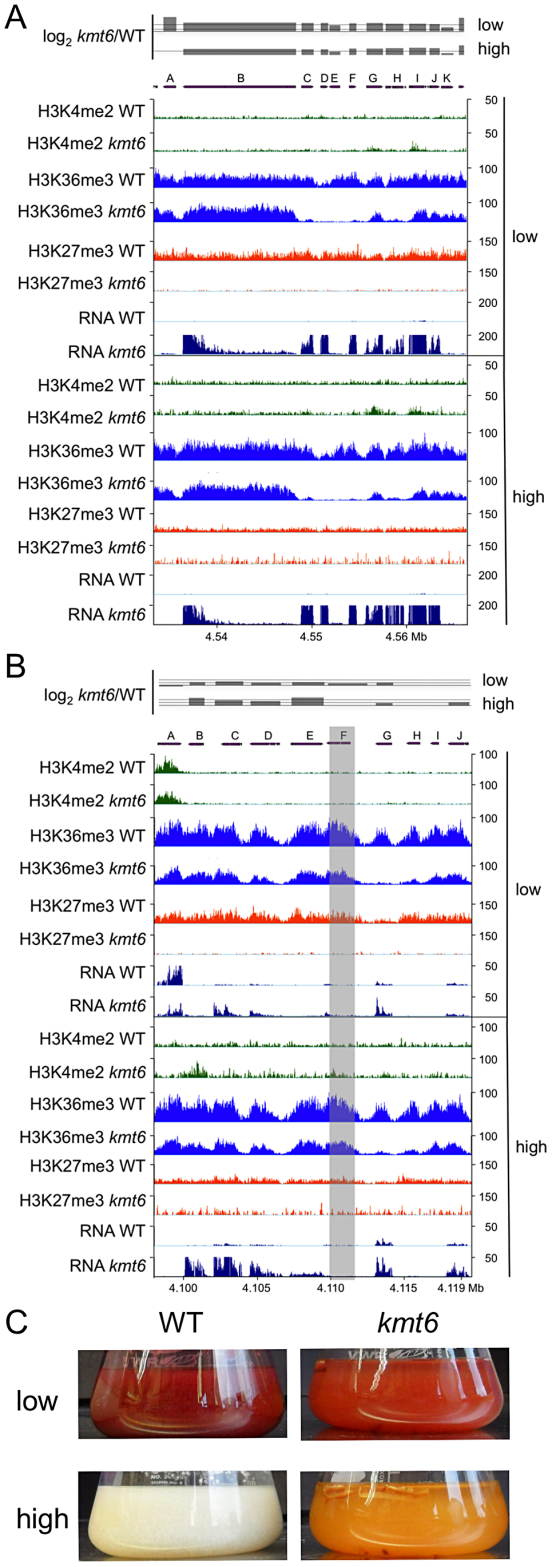
Specific gene clusters are upregulated in *kmt6*. A. All genes in the fusarin C cluster are upregulated (A, FGSG_07797; B, FGSG_07798, *fus1/pks10*; C, FGSG_13222, *fus2*; D, FGSG_13223, *fus3*; E, FGSG_07800, *fus4*; F, FGSG_07801, *fus5*; G, FGSG_07802, *fus6*; H, FGSG_07803, *fus7*; I, FGSG_07804, *fus8* cytochrome P450; J, FGSG_07805). B. The carotenoid cluster is derepressed in *kmt6*. A. Histone modification state and expression of carotenoid biosynthetic genes (A, FGSG_03063, ammonium permease; B, FGSG_03064, rhodopsin; C, FGSG_03065, *al-1/carB* phytoene dehydrogenase; D, FGSG_03066, *al-2/carRA* phytoene synthase; E, FGSG_03067, *cao-1*/*carX* carotenoid dioxygenase; F, FGSG_03068, *carR* transcriptional regulator; G, FGSG_03069, dihydrodipicolinate synthetase; H, FGSG_12520, RING finger protein; I, FGSG_03070; J, FGSG_03071, FAD-dependent oxidoreductase). Genes *al-1*, *al-2* and FGSG_03069 are most strongly upregulated, and the transcription factor *carR* is 3-fold induced in low nitrogen (log_2_ = 1.63), and 1.5-fold induced in high nitrogen (log_2_ = 0.6). In A and B, the log_2_-fold change in expression has a solid line for the x-axis, and dashed lines at 2 (4-fold increase) and 6 (64-fold increase). Only statistically significant values are shown (*p*<0.001). C. Lack of KMT6 induces production of carotenoids and accumulation in the culture liquid at low but especially high nitrogen levels. WT and kmt6 were incubated for 7 days at 28C in liquid ICI with 6 mM or 60 mM NH_4_NO_3_.

To illustrate effects of *kmt6* at the gene level, changes in histone modifications and expression are shown for two representative SM gene clusters (Fig. 8). The fusarin C (f*us*) mycotoxin cluster was induced in *kmt6* when H3K27me3 was lost, yet H3K4me2 enrichment was barely above background levels (Fig. 8A). Nearly every gene in the *fus* cluster was induced more than 64-fold in both low and high nitrogen. Both *fus6* (G, FGSG_07803), encoding a transporter, and *fus8* (I, FGSG_07804), encoding a cytochrome P450, acquired small peaks of H3K4me2. The genes that had increased expression also lost enrichment of H3K36me3. Overexpression of the fusarin C cluster genes can cause production of various fusarins [40].

The carotenoid cluster (*car*) encodes the enzymes required to synthesize the pigments neurosporaxanthin and torulene [41], resulting in the orange *kmt6* culture liquid and mycelium grown on plates (Fig. 8 B and C). The transcription factor gene *carR* (F) was induced 3-fold in low nitrogen only, but in both high and low nitrogen the biosynthetic enzymes *carO* (B), *carB* (C), *carRA* (D), and *carX* (E) were induced more than 4-fold in *kmt6*. The *carO* gene acquired some H3K4me2 in high nitrogen, but none of the other genes in the cluster were enriched for H3K4me2. The reduction in H3K36me3, seen in the other examples at genes with increased expression, was most pronounced in the *car* cluster at gene G (FGSG_03069, dihydrodipicolinate synthetase). WT cultures of *F. graminearum* produce multiple dark red pigments in nitrogen limiting conditions, but expression is repressed under high nitrogen conditions in the dark [42]. In contrast, the culture supernatant of *kmt6* was reproducibly bright red in low nitrogen, and turned bright orange in high nitrogen (Fig. 8C).

The predicted secretome [43], composed of putative effector proteins required for virulence and also including plant cell wall degrading enzymes, phytotoxins and antifungals, is largely encoded in the same regions of the genome where we mapped the SM cluster genes, and secreted protein genes are overwhelmingly enriched for H3K27me3 (Table S2). In summary, the partially overlapping sets for SM gene clusters and secretome genes are localized to subtelomeric regions, enriched for H3K27me3, and induced in *kmt6*. Many genes with unknown function in the same regions follow these general trends, and we predict that they also function in pathogenicity or niche adaptation. The newly found ability to express many of these genes in a single mutant and *in vitro* represents an important step forward in the functional characterization of natural products, not just in *F. graminearum* but also in a wide variety of additional species.

## Discussion

### Distribution Of Histone Modifications In *F. Graminearum* Chromatin

One of our goals is to understand the genome organization of *F. graminearum* and the various types of chromatin associated with specific regions. To this end we carried out ChIP-seq with antibodies against di- or trimethylated H3K4 (H3K4me2/3) as proxies for nucleosomes that are associated with active chromatin segments, or H3K27me3 for facultative heterochromatin. H3K4me2/3 and H3K27me3 were found in mutually exclusive, gene-rich blocks of the genome, as reported for mammals [44]. The patterns of histone modifications we observed differ from published reports of genome-wide patterns in other fungi. As mentioned above, both budding and fission yeast lack KMT6 homologs to generate H3K27me3 [21]. The best-studied filamentous fungus, *N. crassa*, has H3K4me2 in nearly all gene-rich chromatin, but large, heterochromatic, gene-poor, repeat-rich blocks near telomeres and centromeres are enriched with the silencing H3K9me3 mark [16],[45],[46]. In *Neurospora*, H3K27me3 is found in smaller blocks that cover genes and heterochromatic repeats close to telomere ends and these are exclusive of H3K9me3 [16],[17]. Overlap with H3K4me2 distribution has not been studied in detail in *Neurospora*. Various species of *Aspergillus* seem to use H3K9me3 to silence subtelomeric gene clusters [47], although genome-wide studies have not been published. All *Aspergillus* species lack clear KMT6 homologues (our data and [21]). This suggests that different clades of filamentous fungi make use of different chromatin-based regulatory systems to control SM gene clusters.

We assessed distribution of histone modifications across the “average” gene. Overall, H3K27me3, H3K4me2/3 and H3K36me3 distributions across genes and proximal promoters were similar to previous results from plants, fungi and animals: H3K4me2/3 were most pronounced near the 5′ end of genes, while H3K36me3 was found more enriched near the 3′ end of genes. In *N. crassa* H3K4me2 is enriched uniformly throughout the gene body [48], while H3K4me3 is enriched in 5′ ends of genes [45] and H3K36me3 is enriched in 3′ ends of genes [48]. Our findings agree with published animal studies [7, with the exception of the vast extent of H3K36me3 enrichment in nearly all genes. The H3K36me3 KMT is thought to function in association with elongating RNAP and only modify actively transcribing genes [15]. However, in *F. graminearum* nearly all genes are significantly enriched for H3K36me3, though almost half of all genes are not expressed in WT. Preferential enrichment of activating marks in exons compared to introns observed in *Caenorhabditis elegans* [49] was not found in *F. graminearum*. H3K36me3 can regulate mismatch repair by interactions with human MutS homologues [50], suggesting additional roles for H3K36me3 beyond transcription elongation. To uncover the meaning of the strong H3K36me3 enrichment will require additional studies. Nevertheless, the overall patterns of enrichment for H3K4 and H3K36 methylation across genes were not altered in *kmt6*, suggesting that there is little feedback into genic distribution of these marks by H3K27me3 or other putative activities of KMT6. Enrichment of H3K4me2/3 at certain genes was altered in the absence of H3K27me3, suggesting that nucleosomes with activating marks are incorporated into chromatin in the absence of the silencing H3K27me3 modification.

### Correlation Of Histone Modifications And Transcription

While 627 genes were enriched with H3K27me3 and H3K4me2/3, a hallmark of “bivalent” regions [6], most of these genes had strong H3K4me2/3 enrichment in combination with H3K27me3 enrichment just above background levels. Examination of genes with strong enrichment for H3K4me2/3 and H3K27me3 did not reveal a functional enrichment for any particular group of genes. Thus, our data suggest that bivalent promoters or genes can occur in *F. graminearum* but additional work on the biological function of these regions is needed to confirm results from our genome-wide analyses. In *Arabidopsis thaliana*, expressed genes are associated with H3K4me3 and repressed genes are associated with H3K27me3, but 13% of genes are marked with both modifications, including genes with tissue-specific expression and for some TFs that are poised for transcription [51]. Many individual genes, however, are thought to have one or the other modification, where the observed bivalency may have been caused by fractions of mixed nuclei [51], something we cannot exclude for *F. graminearum*.

There were 331 genes enriched with H3K27me3 that showed at least twofold decrease in expression in *kmt6*. These include all four ammonium transporters, three out of six nucleoside permeases, amino acid and oligopeptide permeases, and hydrolases. As recently discussed for *Drosphila* [52], *kmt6* and the genes for the other PRC2 components showed significant H3K27me3 enrichment while they were expressed. The observed decrease in expression of some genes upon loss of H3K27me3 may be due to indirect effects, but it remains possible that H3K27me3 is in some cases required for transcription, which will be subject to further investigation.

Lack of H3K27me3 resulted in activation of ∼14% of all predicted or known genes (1,780/13,354) that were silent in WT. It remains to be seen how many genes are activated directly (e.g. by virtue of “poised” promoters) and how many are activated indirectly (e.g. by involving additional *cis*- or *trans*-acting factors that are controlled by KMT6). Many genes (2,720/13,354 or ∼20% of the genome) remain silent even in the absence of H3K27me3, and 2,575 silent genes possessed none of the investigated modifications. This suggests that while transcription may be the default state in the absence of H3K27me3 regulation for many genes, additional activating factors may be required or some genes are subject to multiple layers of repression.

In budding yeast, H3K4me2 is found in all euchromatic genes, and H3K4me3 is found in actively transcribing or recently transcribed genes [14],[53]. While our study does not address issues of RNA stability, it is likely that many actively transcribing genes in *F. graminearum* lack H3K4me3 under our conditions, are associated with H3K4me2 or even with unmodified H3K4. Our results suggest that “activating” histone modifications are not absolutely required for transcription and that their deposition at transcribed regions is slow and perhaps a secondary event to transcription.

Taken together our results suggest a testable model in which absence of the silencing mark H3K27me3 removes an immediate block to transcription, allowing access to promoters by the basal transcription machinery or completion of the initiation phase of transcription by pre-assembled, or “poised”, transcription machineries on promoters. It appears that activating histone modification marks, such as H3K4me2/3 are only much later, if ever, deposited on these actively transcribing regions, as our growth experiments were carried out over several days. Curiously, the appearance of H3K4me2 appears to correlate with a reduction in H3K36me3 modification, even though all previous data supports the acquisition of both marks before or during transcription [14],[15],[53],[54].

### Production Of Secondary Metabolites, “Epigenetic Engineering” And Genome Organization

Pigment production was very much altered in *kmt6*; the exact pigment profile of *kmt6* grown under various conditions is the subject of an ongoing study (K.M. Smith, J. Gautschi, L.R. Connolly, M. Freitag, unpublished data). From the *kmt6* expression data, some of it summarized in Table 2, it appears that repression by high nitrogen can be overridden by loss of H3K27me3. Overall, loss of H3K27me3 had more drastic effects on expression of SM gene clusters than the intensely studied regulation by nitrogen. For this study we did not measure concentrations of specific known or unknown metabolites, but previous work from several laboratories suggests that increased transcription from SM clusters by manipulation of nitrogen levels, histone H3K9 acetylation or H3K4 methylation levels results in overproduction of certain metabolites [37],[40],[55],[56].

How exactly linear growth is retarded in *kmt6* may be difficult to ascertain. One possibility is that increased synthesis of pigments, other secondary metabolites and detoxifying enzymes may account for the slower growth of *kmt6*, either indirectly by shifting energy utilization away from primary metabolism or by direct toxic effects mediated by combinations of usually harmless metabolites. For example, the red pigment, aurofusarin, is synthesized by the *aur* gene cluster, which includes the polyketide synthase gene *aur1/pks12* [42],[57]. A Δ*aur1* mutant grew faster and generated more conidia than WT on media inducing aurofusarin production [57], suggesting that costs are incurred by the production of specific metabolites. It remains to be seen how or if H3K27 methylation is altered in *aur1* and similar mutants.

Attempts to activate individual silent clusters for chemical genome mining has largely focused on overexpression of cluster-specific regulators, mostly transcription factors [58],[59],[60] or heterologous expression of partial or complete clusters [61,[62],[63]. A more general approach to activate silent gene clusters involves treatment with inhibitors of DNA or histone modifying enzymes. Silent clusters were activated by histone deacetylase inhibitors or DNA methyltransferase inhibitors in *Cladosporium cladosporioides* [64] and *A. niger* [65], or by cocultivation with other organisms, e.g. bacteria or fungi, to mimic a natural environment [56],[66]. These approaches were successful in inducing a few SM clusters, but they are little different from previous attempts to find the exact culture conditions for expression of specific gene clusters. Another strategy to activate silent clusters is focused on global regulators of secondary metabolism. Mutation of selected histone-modifying enzymes predicted to be global gene regulators, e.g. the histone deacetylase HdaA [67], the CclA component of the H3K4MTase complex [55],[68] and the histone acetyltransferase EsaA [69] proved successful in affecting certain clusters in *Aspergillus*. Individual SM gene clusters are affected in different ways by mutating or overexpressing these enzymes and the effects are not specific to SM clusters.

The most widely studied general regulator is the “Velvet complex”, first identified in *A. nidulans* [70] and later also found in *F. verticillioides* [71], *F. fujikoroi* [72] and *F. graminearum* [73],[74]. This complex consists of the putative transcription factors VeA, VelB and a putative methyltransferase, LaeA [75] and regulates both fungal development and SM production. In *A. nidulans*, the complex inhibits asexual reproduction, promotes sexual development and increases SM production in dark conditions [70]. In *A. fumigatus*, 13 of 22 SM clusters and 20–40% of SM biosynthetic genes were expressed at lower levels in a Δ*laeA* strain compared to WT [76]. The molecular mechanism of this pathway presumably involves the methyltransferase domain of LaeA [76]. LaeA controls protein levels and complex interactions between VeA and its partners [77], a function separable from its role as global regulator of SM gene clusters. The precise function of VeA, VelB, and LaeA in changing transcriptional programs has not yet been determined, but it has been suggested to involve re-programming of the constitutive heterochromatin mark, H3K9me3 [47],[78],[79].

Here we revealed a novel mechanism that links fungal development and SM expression, and that appears at least partially conserved with formation of facultative heterochromatin in plants, *Drosophila* and mammals by generation of blocks of H3K27me3-enriched chromatin. Essentially these blocks generate a “cryptic genome” under normal laboratory culture conditions. There is no indication that this process is dependent on members of the velvet complex. We looked for changes in expression for the “white collar” genes (i.e. the light-sensing complex that controls VeA activity), VeA, VelB, VosA and LaeA, and found no differences in expression between WT and *kmt6*. All of these genes were enriched for H3K4me2 and expressed in both WT and mutant. We did, however, find changes in several predicted LaeA homologs whose functions are still largely unknown. Proteins encoded by these genes were found to interact with *F. graminearum* VeA and named “FgVeA interacting proteins”, or VIP [73]. Conserved VIPs are FgVIP1 (FGSG_07660), FgVIP2 (FGSG_03525), FgVIP3 (FGSG_05685), FgVIP4 (FGSG_03567), FgVIP5 (FGSG_08741), and FgVIP6 (FGSG_03011). All VIP genes were enriched for H3K27me3, and loss of this modification in *kmt6* caused increased transcription. Homologues of VIPs have been studied in *A. nidulans*, where LlmF (LaeA-like methyltransferase) interacts with velvet components and appears to shuttle the complex into the nucleus [80]. Thus it appears possible that LaeA homologs are involved in H3K27me3 regulation.

One wonders why gene family expansions and acquisition of SM clusters occurs preferentially in subtelomeric locations. Subtelomeric regions of *Aspergillus* species contain numerous SM clusters, and based on previous results one would expect to find large H3K9me3-enriched domains in these regions [81],[82] but this remains to be demonstrated. There is evidence, at least in *Magnaporthe* and *Saccharomyces* [83],[84],[85],[86], that subtelomeric regions are more prone to rearrangements than other regions of the genome. Published synteny maps of *F. graminearum*, *F. verticilliodes*, and *F. oxysporum*, as well as our preliminary results from studies with a close cousin of *F. graminearum*, *F. asiaticum* (L.R. Connolly, K.M. Smith, S.-H. Yun, M. Freitag, unpublished data), show that subtelomeric regions are hypervariable between related organisms and accumulate SNP mutations at higher rates than other regions of the genome (Fig. 1). This suggests a model in which H3K27me3 is involved in the regulation of recombination or chromosome rearrangements.

Why are SM genes in clusters? This can be explained by the “selfish cluster” hypothesis [87], at least if horizontal gene transfer is not exceedingly rare. Genes in a cluster are more likely to be transferred as a functional group if acquisition and loss of clusters is adaptive to the organism. Selective advantages to the new host organism, specifically by creation of novel clusters and maintenance of all clusters, however, remains unclear. Initially, uptake of novel DNA would not be dissimilar from invading transposable elements, which tend to be silenced by a combination of H3K9me3 and DNA methylation in *N. crassa* [46],[88]. Further partitioning of the genome into additional chromatin domains by making use of H3K27me3 that eventually results in coordinate regulation is a plausible hypothesis to explain the maintenance of secondary metabolite genes in clusters. One wonders if subtelomeric silencing depends on PcG proteins in other fungi. So far, we only have data for *N. crassa* [16],[17] and several *Fusarium* species, but many important animal and plant pathogens within the ascomycetes have predicted orthologues for PRC2 components.

## Materials And Methods

### Growth Conditions

Strains were grown in liquid YPD to collect vegetative tissue. To generate macroconidia, a small amount of frozen conidia or tissue was inoculated into 50 ml flasks containing CMC medium [89] and shaken at 150 rpm for 3–4 days at room temperature (RT, ∼22C). Conidia were collected by filtration through cheesecloth and stored at −80C in 25% glycerol. For vegetative growth assays strains were inoculated onto YPD (0.3% yeast extract, 1% bacto-peptone, 2% dextrose) or Fusarium Minimal Medium (FMM; [90]) agar plates. Crosses were performed on carrot agar at RT, taking usually ∼10 days. To assay pigment production, tissue was generated from macroconidia by shaking 100 ml cultures at 150 rpm in the dark in DVK medium (3% sucrose, 1.5% corn steep solids, 0.1% (NH_4_)_2_SO_4_, and 0.7% CaCO_3_) for three days, after which 5 ml were used to inoculate 100 ml of liquid ICI medium [91] with 6 mM or 60 mM NH_4_NO_3_ for nitrogen limiting or sufficient conditions, respectively. Cultures were grown at 25C at 150 rpm in the dark and observations made after 3 and 7 days of growth.

### Construction Of *Kmt6* Deletion And Complementation Strains

Replacement cassettes with the selectable hygromycin (Hyg) resistance marker (*hph^+^*), and neomycin/G418 resistance marker (*neo^+^*), encoding hygromycin and neomycin phosphotransferase, respectively, were generated by fusion PCR [92]. The 5′ and 3′ flanking regions of the *kmt6* coding region were amplified from genomic DNA of PH-1 (FGSC9075, FMF1) with primers OMF1936 (5′-TCTTGGATATTGGCCAGCTC-3′) and OMF1930 (5′-GATAAGCTTGATATCGAATTCTTACTTGTGGCTfGCGGCTAATTGATGGCT-3′) or OMF1931 (5′-TGCTATACGAAGTTATGGATCCGAGCTCGTTTGGGCAGAGAAGCTTGAATA-3′) and OMF1937 (5′-GTGGAGGGAAAACTTGGTGA-3′), respectively. The *loxP-neo-loxP* cassette was amplified from pLC13-Tom-loxP-neo-loxP with primers OMF1148 (5′-ACAAGTAAGAATTCGATATCAAGCTTATC-3′) and OMF84 (5′-CGAGCTCGGATCCATAACTTCGTATAGCA-3′). The 5′ and 3′ *kmt6* flanks were fused to the *neo^+^* cassette by PCR with *neo* split marker primers OMF601 (5′-AGGCGATGCGCTGCGAATCGG-3′) and OMF1937 or OMF600 (5′-TTGAACAAGATGGATTGCACG-3′) and OMF1936. PCR-amplified fragments were gel-purified using a Qiaquick gel purification kit.

For transformations, ∼10^7^ PH-1 conidia were inoculated into 100 ml of YPD and allowed to germinate overnight at 28C with shaking at 200 rpm. Mycelia were harvested on cheesecloth and about 1 g (wet weight) was transferred into 20 ml of 1.4 M KCl with 500 mg driselase (Sigma, D8037), 100 mg lysing enzyme (Sigma, L1412), and 1 mg chitinase (Sigma, C6137) and shaken gently at 90 rpm at 28C for 2.5 hrs to induce protoplast formation. The suspension was filtered through Nitex membrane (30 µM) and protoplasts were collected and counted. Transformants were generated by mixing ∼10^7^ protoplasts with 1 µg of *neo^+^* split marker fragments in 500 µl of STC and 30% PEG8000 (4∶1) and incubating at RT for 20 min. An additional 1 ml of 30% PEG was added and the mixture was incubated for another 5 min, after which 2 ml of STC were added and the mixture was combined with 87 ml of recovery medium (RM) and split between six 100 mm Petri dishes for a total 15 ml RM per dish. After 24 hrs at RT, the RM was overlayed with 15 ml RM+200 µg/ml G418. Resistant colonies were picked and purified from single conidia by generating spores in liquid CMC medium. Strains were screened for gene replacements by PCR and Southern analyses.

We generated a strain containing a wildtype *kmt6* allele for complementation analyses (FMF282) by random ectopic insertion into the *kmt6* deletion strain FMF248. We digested pFOLT4R4 [93] with *Cla*I and isolated a 4 kb fragment that contained telomere repeats. This fragment was digested with *Pvu*II for cloning into the *Sma*I site of pBSII SK+ [94], generating pLC14. The *Sal*I *hph* fragment of pCT74 [95] was inserted pLC14 to generate pLC15. The *kmt6* gene was PCR amplified with OMF1936 and OMF1937 and inserted into pCR4-TOPO (Invitrogen) to generate pLC40. The *kmt6* coding region with ∼1 kb 5′ and 3′ flanks was released from pLC40 with *Spe*I and inserted into the *Spe*I site of pLC15 to generate pLC41. This plasmid was transformed into the Δ*kmt6* strain (FMF248) as described above and Hyg^+^ transformants were screened for integration by Southern analyses.

### Protoplast Fusions

Approximately 5×10^6^ protoplasts of FMF 225 (heterokaryotic *kmt1^+^/kmt1*) and FMF 248 (*kmt6::neo^+^*) were mixed and plated at a density of 2.5×10^6^ protoplasts per dish on RM with 100 µg/ml Hyg, and 100 µg/ml G418. After one week, a plug from a selected colony was transferred onto YPD agar with 200 µg/ml Hyg and 200 µg/ml G418. Plugs from this heterokaryon were transferred to carrot agar for selfings. Selfings and crosses were performed as described previously [96], with minor modifications.

### Growth On Wounded Tomato Fruits

To assay colonization of tomato fruits, ripe organically grown “Roma” tomatoes (Denison Farms, Corvallis, OR) were surface-sterilized by gently wiping fruit with 95% ethanol, as described previously [32]. A small region of the epidermis (∼10 mm^2^) was peeled back and the wound was infiltrated with 10 µl of spore suspensions containing ∼1,000 conidia (1×10^5^ conidia/ml). Fruits were incubated at 28C above water reservoirs, increasing humidity.

### Southern Analyses

Genomic DNA was isolated according to a previously published method [97], digested with *Hind*III, and blotted as described elsewhere [98].

### Western Analyses

Tissue for histone extractions was generated by inoculating ∼10^7^ macroconidia into 100 ml YPD and shaking at 200 rpm at 28C for 2 days. Mycelia were harvested by filtration, frozen in liquid nitrogen, and ground to a fine powder with a mortar and pestle. Histones were acid-extracted as previously described [99]. Approximately 10 to 20 µg of total protein per lane were analyzed by SDS-PAGE. Proteins were transferred to PVDF membrane and blotted using standard procedures [100]. Primary antibodies for westerns were Millipore 07-030 for H3K4Me2, Active Motif 39159 for H3K4me3, abcam ab8898 and Active Motif 39161 for H3K9Me3, and abcam ab9050 for H3K36Me3. We used four different antibodies to detect H3K27me3, Active Motif 39535, abcam ab6002 and ab6147, and Active Motif 39155 (which resulted in high background). Secondary antibodies were HRP-conjugated goat anti-rabbit (Pierce 31460) or HRP-conjugated goat anti-mouse (Invitrogen 62-6520).

### Chromatin Immunoprecipitation (chip) And High-Throughput Sequencing

ChIP was carried out on mycelia generated by growing conidia in 100 ml DVK medium for 3 days, transferring 5 ml of the suspension to 100 ml ICI medium supplemented with 6 mM or 60 mM NH_4_NO_3_ for nitrogen limiting or sufficient conditions, respectively, and shaken at 200 rpm for 48 to 72 hrs in the dark at 28C. ChIP methods were essentially as described previously [45],[101]. Strains and antibodies used for ChIP are listed in Table S1. DNA obtained by ChIP was end-repaired and ligated to adapters as described elsewhere [102]; adapter barcodes are listed in Table S1). Fragments (300 to 500 bp long) were gel-purified and amplified by 21–24 cycles of PCR with Phusion polymerase (Finnzymes Oy, NEB) and Illumina PCR primers [102]. Libraries were sequenced on an Illumina GAII and processed with RTA1.8 and CASAVA1.7 or on a HiSeq2000 genome analyzer and processed with CASAVA1.8. Wild-collected strain 00-676 [28] was re-sequenced to identify SNPs, which were called with MAQ [103].

### Rna Isolation And High-Throughput Sequencing (“Rna-Seq”)

Total RNA was isolated from aliquots of the same tissue that was used for ChIP by a previously described method [104], and mRNA was isolated using a Poly(A)Purist MAG kit (Ambion). We removed DNA by treatment with RNase-free DNAase (Qiagen), followed by column clean-up according to manufacturer's instructions. We used Illumina TruSeq RNA Sample Preparation kits to make RNA-seq libraries; cDNA was sequenced on an Illumina HiSeq2000 genome analyzer.

### Short Read Mapping And Data Analysis

ChIP-seq reads were sorted by adapter and adapter sequences were removed, then quality scores were converted to Sanger format with the MAQ sol2sanger command [103] if needed (depending on Illumina pipeline output). HTS data from ChIP- and RNA-seq were submitted to the NCBI GEO database (accession number: GSE50689). Fastq files were used as input for BWA [105] and aligned to a reformatted assembly 3 of the *F. graminearum* genome (http://www.broadinstitute.org/annotation/genome/fusarium_group/MultiHome.html), i.e. supercontigs were assembled into chromosomes and separated by 20 kb of Ns as placeholders for unassembled reads to match the Broad Institute v3 chromosome assembly. Sam-formatted alignment files from BWA were converted to bam format, sorted, and indexed with samtools [106] for viewing in the gbrowse2 genome browser [107]. Data are accessible at http://ascobase.cgrb.oregonstate.edu/cgi-bin/gb2/gbrowse/fgraminearum_public/. Adapter-trimmed RNA-seq reads were mapped with Tophat [108] with options -a 5 -m 1 -i 30 -I 2000 and processed in the same way as BWA output with samtools. Cufflinks was used to quantify gene expression values as reads per kilobase of exon per million reads (RPKM), and cuffdiff was used to identify differentially expressed genes between samples [36],[109]. Figures showing global RNA expression and comparison between samples were generated in R with CummeRbund [36]. Heatmaps were generated in R with pheatmap (http://cran.r-project.org/web/packages/pheatmap/pheatmap.pdf). In total, 1,628 genes in fungidb.org were found to be associated with GO term “0044238, primary metabolic process”. Of these, 1,389 had significant data from cuffdiff and were used to generate heatmaps. Secondary metabolite genes were identified from the Broad Institute database. K-means clustering with 12 centers was done in R with the default Hartigan and Wong algorithm [110] to generate “clustered” heatmaps. We found 113 cytochrome P450 genes by searching for PFAM domain “PF00067: p450” at fungidb.org, and 77 of these genes had significant values from cuffdiff and were included in the heatmap. NRPS [111] and PKS [42] genes were previously described, but we use current FGSG numbers here [112]. A near complete secretome gene set has been described [43]. The list of transcription factors from a comparative study of three *Fusarium* species was used [27]. Transcription factors required for perithecial development were previously identified [113].

## Supporting Information

Figure S1 Domain structure of KMT6 and alignment of conserved domains. A. *Fusarium graminearum* KMT6 is compared to SET-7 from *Neurospora crassa* (EAA35807), *Drosophila melanogaster* E(Z) (P42124.2) and *Homo sapiens* EZH2 (Q15910.2). Domains were found with the ProSite tool on ExPASy (http://prosite.expasy.org/). While there is little overall sequence similarity between metazoan and fungal KMT6 proteins, the CXC (pre-SET) and SET domains are well conserved. The CXC domains extend from 606–723, 900–1023, 518–619 and 503–605 amino acids, the SET domains from 751–871, 1051–1171, 625–745 and 611–731 aa in the Fusarium, Neurospora, Drosophila and human protein, respectively. B. Alignment of the CXC and SET domains of the proteins shown above. Certain residues (bold black type) are conserved in most CXC and SET domains in bona fide histone methyltransferases (Zhang et al, Cell, 2002), while some residues in all four proteins shown are found more often in E[Z]/EZH2 or KMT6 proteins (bold red type). All four SET domains are 120 aa long and contain residues that are involved in formation of a “pseudoknot”, and have the invariant tyrosine essential for SAM binding and catalysis (indicated by #). Identical residues in all four proteins are indicated by and asterisk (*), while colons (:) and periods (.) indicate conservative and less conservative changes in the primary protein sequence.
(TIF)

Figure S2 Validation of *kmt6* mutant. A. Southern analyses show no signal in the *kmt6* mutant (FMF248) when probed with the *kmt6* coding region, which usually detects a 2.5 kb fragment (left panel), while the *neo^+^* gene (1.7 kb) is present (center panel). The reverse is seen in a wild type strain (WT). The mutant *kmt6* strain was transformed with *kmt6^+^* on a plasmid with the *hph^+^* marker, which resulted in complementation by ectopic integration of several *kmt6* copies (*kmt6 kmt6^+^-hph^+^*; left panel; FMF282) and presence of *hph* (right panel; the major band is at 4.6 kb). A control strain transformed with the *hph^+^* plasmid only (*kmt6 hph^+^*) shows signal when probed with *hph* but not when probed with *kmt6*. The *neo^+^* gene is retained in both transformants. There is some cross hybridization between the *neo* and *hph* regions as both probes contain part of the *A. nidulans trpC* promoter. Genomic DNA from each strain was left undigested (U) or digested with *Hin*dIII (H), separated through a 0.8% agarose gel, blotted to nylon membrane and probed with ^32^P-labelled probes. Major bands match expected sizes from maps of the *kmt6* region; the complemented strain carries a multicopy ectopic insertion of the whole plasmid but the major band matches the *kmt6* coding region. B. Western analyses with acid-extracted histones from WT (FMF1) and *kmt6* (FMF248) show absence of H3K27me3, but unchanged levels of H3K9me3, H3K4me2, and H3K36me3. Approximately 10 to 20 µg of acid-extracted histones or commercial calf thymus histones (calf; Sigma) were loaded. (TIF)

Figure S3 H3K27me3 occurs in large blocks. H3K27me3 (orange) is mutually exclusive of H3K4me2 (green) and -me3 (blue), which are found in the same regions of chromosomes 2 to 4. Only minor differences were observed between strains grown in low and high nitrogen. H3K27me3 is completely lost in the *kmt6* mutant but H3K27me3 distribution matches that of WT in a complemented strain (*kmt6^+^*). (TIF)

Figure S4 Growth of WT (left) and *kmt6* (right) on wounded tomatoes at five days after inoculation. While *F. graminearum* primarily causes symptoms on cereal plants, it can also cause fruit rot on green and ripe tomato [32]. We inoculated WT and *kmt6* (FMF248) on ripe organically grown tomatoes whose epidermis had been peeled back at the point of inoculation. Representative outcomes from three independent growth experiments with three fruits of each WT and *kmt6* are shown here. While the WT strain colonized the fruit successfully within five days after inoculation and by 14 days covered more than half of the fruit, the *kmt6* inoculum did not survive (re-isolations were unsuccessful), though a few spores were able to germinate. (TIF)

Figure S5 Global transcriptional analysis and correlation with histone modifications. Biological replicates are highly correlated and pairwise comparisons of FPKMs for each gene, WT *vs. kmt6* and low *vs.* high nitrogen for each strain shows global changes in gene expression. The distribution of FPKM scores for all four conditions is shown. (TIF)

Table S1 HTS statistics. Reads obtained from ChIP-seq and RNA-seq experiments, numbered by unique ID (HTS#), with WT (FMF1), *kmt6* (FMF248) and the complemented *kmt6^+^* (FMF282) strains under low and high nitrogen availability ([N]) are tabulated, and antibodies used for ChIP are shown. Antibody sources and catalog numbers are shown (Active Motif, AM; Millipore, M; abcam, ab). Barcoded Illumina libraries were generated by ligation to custom-made adapters (five nucleotides; [102]) or with TruSeq kits (six nucleotides; Illumina) and subjected to 58-nt single-end sequencing. Reads were parsed and mapped as described in the Materials and Methods; the percentage of mapped reads (%) indicates quality of the ChIP- or RNA-seq library. (DOCX)

Table S2 Genes included in specific clusters depending on correlation of H3K27me3 enrichment and expression in WT and kmt6. Only clusters discussed in the manuscript are listed here. (DOCX)
