# Supplementary figures and images for: The Fusarium graminearum Histone H3 K27 Methyltransferase KMT6 Regulates Development and Expression of Secondary Metabolite Gene Clusters
Source: PLoS Genet. 2013 Oct 31;9(10):e1003916. doi: 10.1371/journal.pgen.1003916 (PMC3814326; doi:10.1371/journal.pgen.1003916)

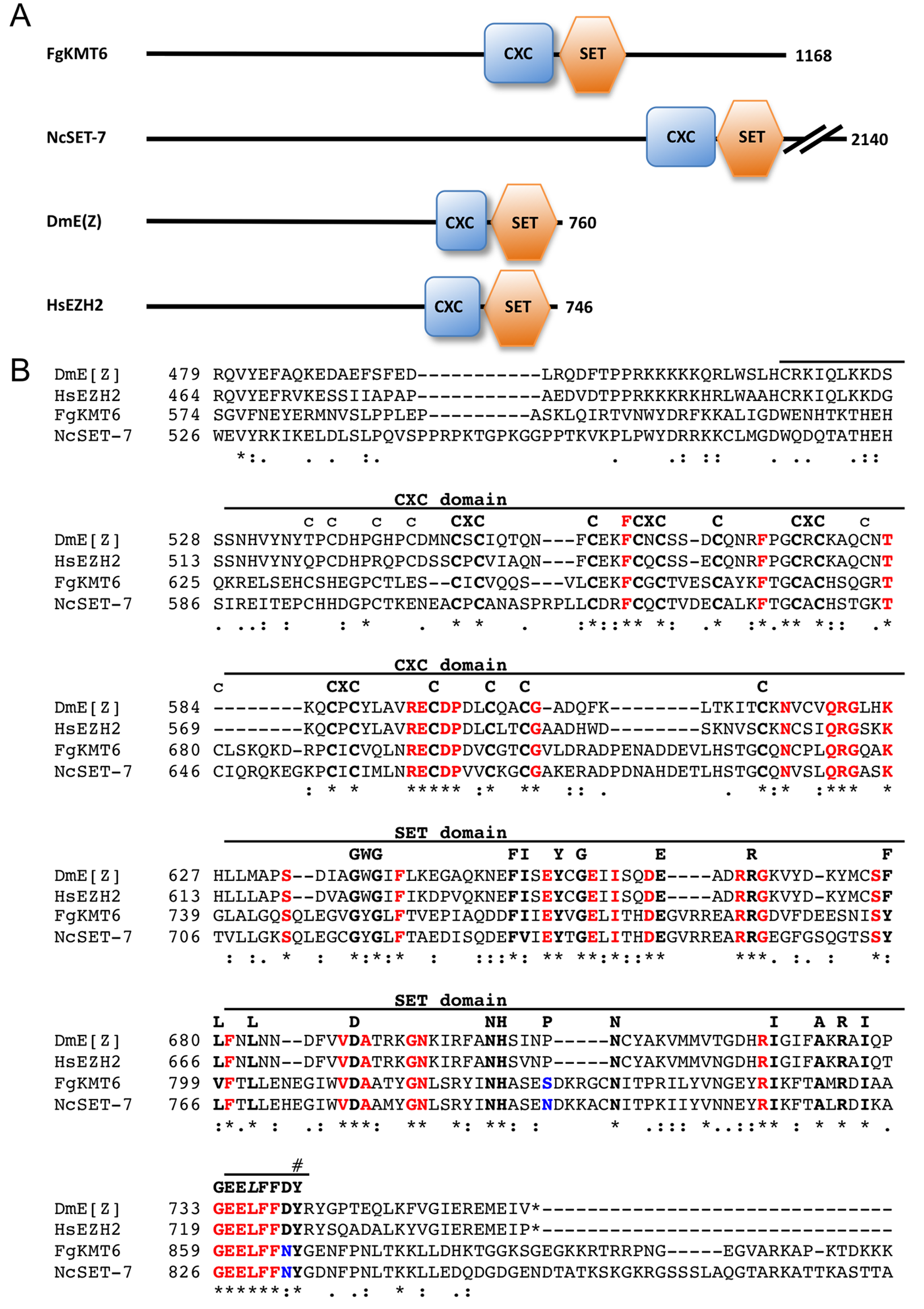

Supplement: Figure S1 — Domain structure of KMT6 and alignment of conserved domains. A. Fusarium graminearum KMT6 is compared to SET-7 from Neurospora crassa (EAA35807), Drosophila melanogaster E(Z) (P42124.2) and Homo sapiens EZH2 (Q15910.2). Domains were found with the ProSite tool on ExPASy (http://prosite.expasy.org/). While there is little overall sequence similarity between metazoan and fungal KMT6 proteins, the CXC (pre-SET) and SET domains are well conserved. The CXC domains extend from 606–723, 900–1023, 518–619 and 503–605 amino acids, the SET domains from 751–871, 1051–1171, 625–745 and 611–731 aa in the Fusarium, Neurospora, Drosophila and human protein, respectively. B. Alignment of the CXC and SET domains of the proteins shown above. Certain residues (bold black type) are conserved in most CXC and SET domains in bona fide histone methyltransferases (Zhang et al, Cell, 2002), while some residues in all four proteins shown are found more often in E[Z]/EZH2 or KMT6 proteins (bold red type). All four SET domains are 120 aa long and contain residues that are involved in formation of a “pseudoknot”, and have the invariant tyrosine essential for SAM binding and catalysis (indicated by #). Identical residues in all four proteins are indicated by and asterisk (*), while colons (:) and periods (.) indicate conservative and less conservative changes in the primary protein sequence. (TIF) [file pgen.1003916.s001.tif]

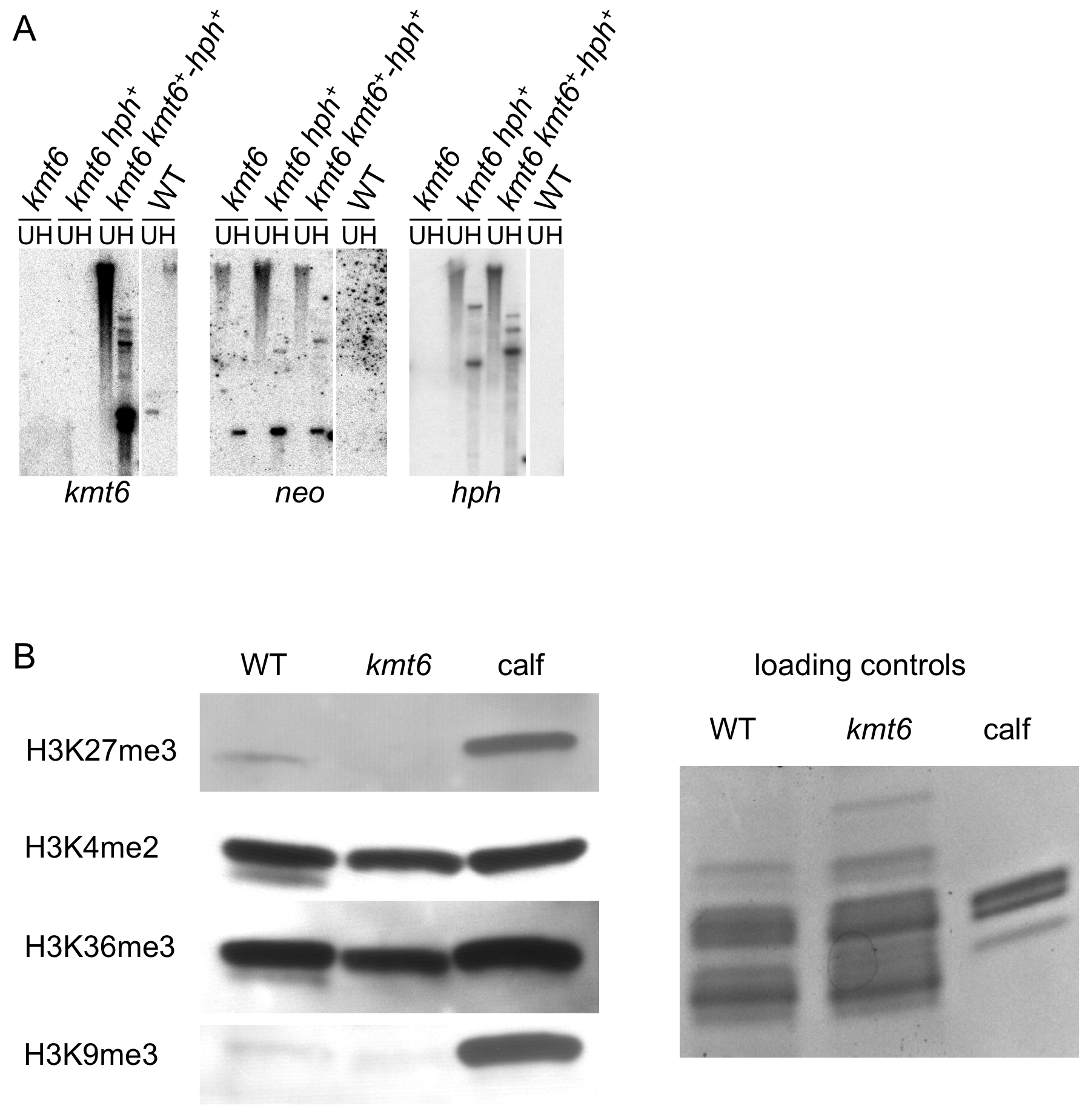

Supplement: Figure S2 — (TIF) [file pgen.1003916.s002.tif]

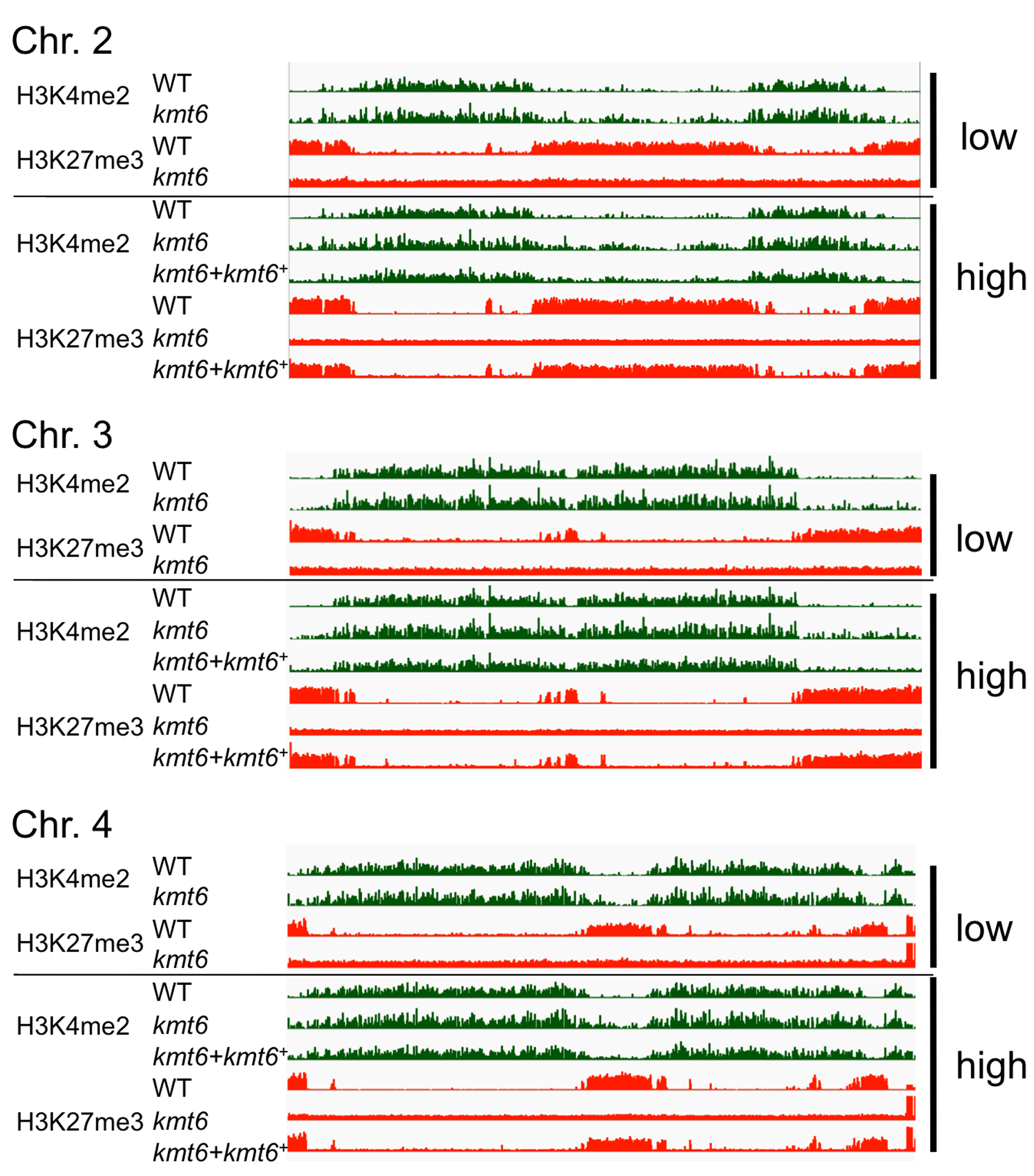

Supplement: Figure S3 — (TIF) [file pgen.1003916.s003.tif]

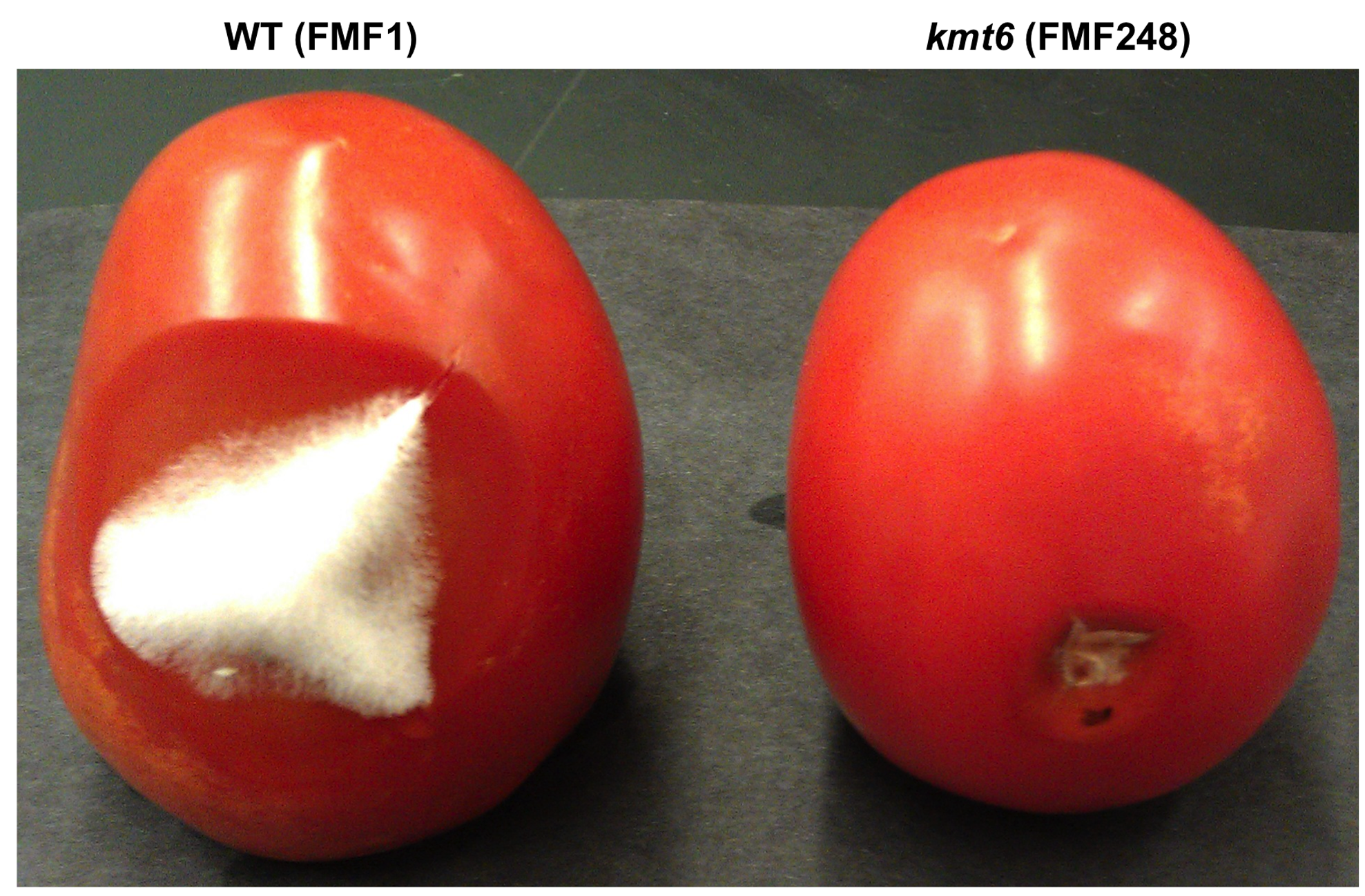

Supplement: Figure S4 — (TIF) [file pgen.1003916.s004.tif]

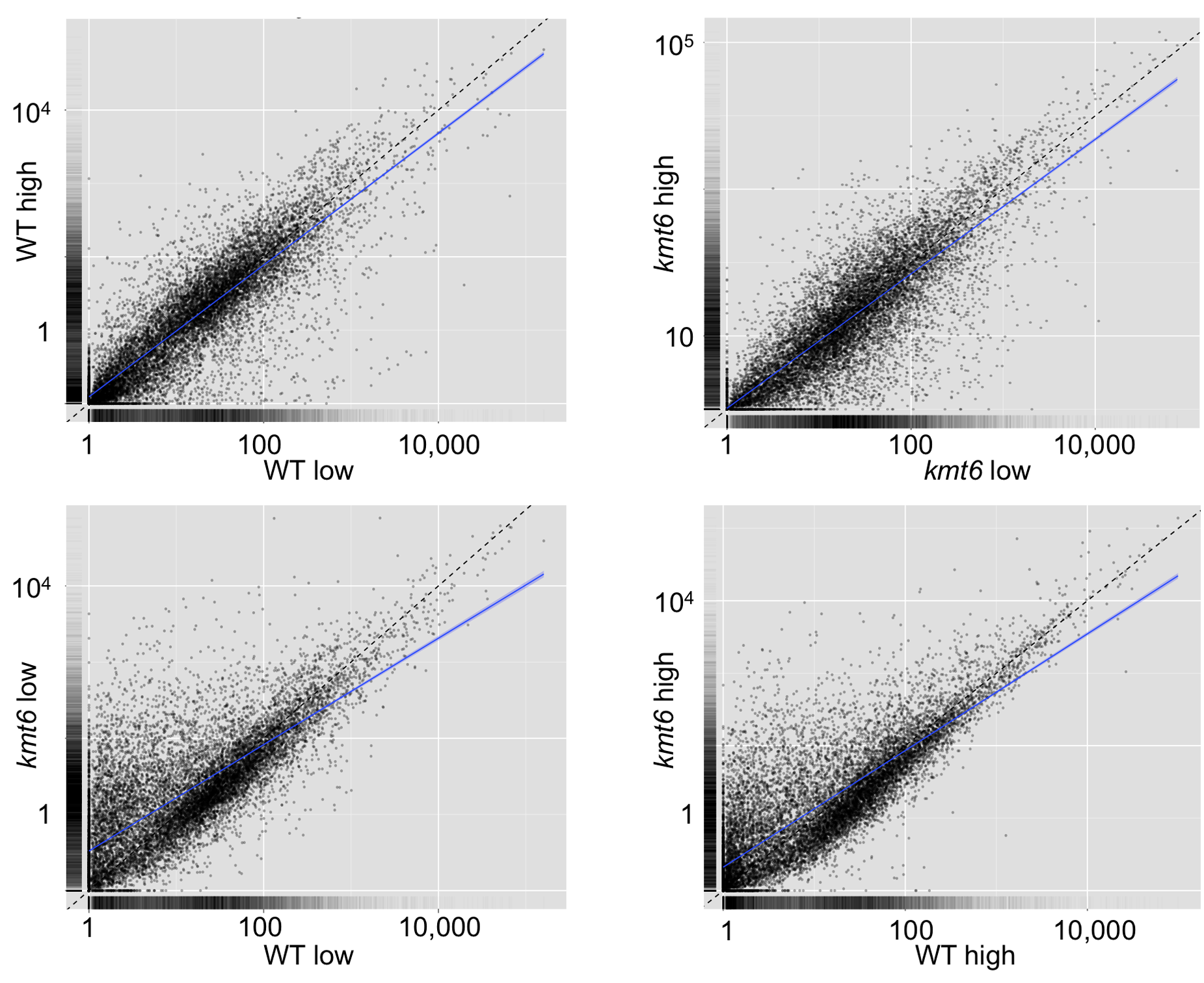

Supplement: Figure S5 — (TIF) [file pgen.1003916.s005.tif]
