# Supplementary material for: The Fusarium graminearum Histone H3 K27 Methyltransferase KMT6 Regulates Development and Expression of Secondary Metabolite Gene Clusters
Source: PLoS Genet. 2013 Oct 31;9(10):e1003916. doi: 10.1371/journal.pgen.1003916 (PMC3814326; doi:10.1371/journal.pgen.1003916)
Supplement: Table S1 — (DOCX) [file pgen.1003916.s006.docx]

**Supplemental Table 1: HTS statistics.** Reads obtained from ChIP-seq and RNA-seq experiments, numbered by unique ID (HTS#), with WT (FMF1), *kmt6* (FMF248) and the complemented *kmt6^+^* (FMF282) strains under low and high nitrogen availability ([N]) are tabulated, and antibodies used for ChIP are shown. Antibody sources and catalog numbers are shown (Active Motif, AM; Millipore, M; abcam, ab). Barcoded Illumina libraries were generated by ligation to custom-made adapters (five nucleotides; {Pomraning, 2012 #12099}) or with TruSeq kits (six nucleotides; Illumina) and subjected to 58-nt single-end sequencing. Reads were parsed and mapped as described in the Materials and Methods; the percentage of mapped reads (%) indicates quality of the ChIP- or RNA-seq library.

| **HTS#** | **Strain** | | **[N]** | | | **Antibody** | **Source** | **Adapter** | **Parsed reads** | **Mapped reads** | **%** |  |
| --- | --- | --- | --- | --- | --- | --- | --- | --- | --- | --- | --- | --- |
| **a. ChIP-seq** | | |  | |  |  |  |  |  |  |  | |
| HTS480 | WT | | low | | | H3K27me3 | AM 39155 | ACGTT | 14983721 | 13749349 | 91.76 |  |
| HTS482 | WT | | high | | | H3K27me3 | AM 39155 | AGTCT | 6871011 | 6483819 | 94.36 |  |
| HTS715 | WT | | high | | | H3K27me3 | AM 39155 | TGACCA | 1617620 | 413493 | 25.56 |  |
| HTS717 | WT | | high | | | H3K27me3 | AM 39155 | GCCAAT | 5110400 | 3403943 | 66.61 |  |
| HTS633 | WT | | low | | | H3K36me3 | ab 9050 | CTTGTA | 37931109 | 33714264 | 88.88 |  |
| HTS635 | WT | | high | | | H3K36me3 | ab 9050 | AGTTCC | 22204706 | 19444707 | 87.57 |  |
| HTS479 | WT | | low | | | H3K4me2 | M 07-030 | CGTAT | 15545387 | 14531975 | 93.48 |  |
| HTS481 | WT | | high | | | H3K4me2 | M 07-030 | GTACT | 12851288 | 12462277 | 96.97 |  |
| HTS714 | WT | | high | | | H3K4me2 | M 07-030 | CGATGT | 1513114 | 1091579 | 72.14 |  |
| HTS716 | WT | | high | | | H3K4me2 | M 07-030 | ACAGTG | 8569375 | 7708159 | 89.95 |  |
| HTS632 | WT | | low | | | H3K4me3 | ab 8580 | CAGATC | 22563631 | 19280032 | 85.45 |  |
| HTS634 | WT | | high | | | H3K4me3 | ab 8580 | AGTCAA | 24505737 | 19958065 | 81.44 |  |
| HTS404 | *kmt6* | | low | | | H3K27me3 | AM 39155 | AGTCT | 5306066 | 4502036 | 84.85 |  |
| HTS406 | *kmt6* | | high | | | H3K27me3 | AM 39155 | CTAGT | 19956498 | 17570575 | 88.04 |  |
| HTS484 | *kmt6* | | low | | | H3K27me3 | AM 39155 | CTAGT | 1617530 | 1077424 | 66.61 |  |
| HTS486 | *kmt6* | | high | | | H3K27me3 | AM 39155 | CTAGT | 11733060 | 6942821 | 59.17 |  |
| HTS637 | *kmt6* | | low | | | H3K36me3 | ab 9050 | GTGAAA | 25831061 | 22056663 | 85.39 |  |
| HTS639 | *kmt6* | | high | | | H3K36me3 | ab 9050 | CCGTCC | 28159483 | 25295337 | 89.83 |  |
| HTS403 | *kmt6* | | low | | | H3K4me2 | M 07-030 | GTACT | 21742074 | 21237210 | 97.68 |  |
| HTS405 | *kmt6* | | high | | | H3K4me2 | M 07-030 | TACGT | 16450325 | 16043750 | 97.53 |  |
| HTS483 | *kmt6* | | low | | | H3K4me2 | M 07-030 | TACGT | 6214153 | 5782922 | 93.06 |  |
| HTS485 | *kmt6* | | high | | | H3K4me2 | M 07-030 | AGTCT | 21119437 | 20375679 | 96.48 |  |
| HTS718 | *kmt6* | | high | | | H3K4me2 | M 07-030 | CAGATC | 2957523 | 2449750 | 82.83 |  |
| HTS636 | *kmt6* | | low | | | H3K4me3 | ab 8580 | ATGTCA | 20000000 | 15919121 | 79.6 |  |
| HTS638 | *kmt6* | | high | | | H3K4me3 | ab 8580 | GTCCGC | 24214304 | 18693790 | 77.2 |  |
| HTS723 | *kmt6+* | | high | | | H3K27me3 | AM 39155 | CCGTCC | 10600656 | 7052247 | 66.53 |  |
| HTS725 | *kmt6+* | | high | | | H3K27me3 | AM 39155 | GTGAAA | 26025127 | 16585513 | 63.73 |  |
| HTS722 | *kmt6+* | | high | | | H3K4me2 | M 07-030 | ATGTCA | 22942098 | 19827274 | 86.42 |  |
| HTS724 | *kmt6+* | | high | | | H3K4me2 | M 07-030 | GTCCGC | 5147209 | 3644138 | 70.8 |  |
| **b. RNA-seq** | |  | |  | |  |  |  |  |  |  |  |
| HTS582 | WT | | low | | | N/A | N/A | TGACCA | 95292016 | 95292016 | 100 |  |
| HTS583 | WT | | low | | | N/A | N/A | ACAGTG | 150319242 | 150319242 | 100 |  |
| HTS584 | WT | | high | | | N/A | N/A | GCCAAT | 162375439 | 162375439 | 100 |  |
| HTS585 | WT | | high | | | N/A | N/A | CAGATC | 121229746 | 121229746 | 100 |  |
| HTS586 | *kmt6* | | low | | | N/A | N/A | AGTTCC | 125146272 | 125146272 | 100 |  |
| HTS587 | *kmt6* | | low | | | N/A | N/A | ATGTCA | 142257430 | 142257430 | 100 |  |
| HTS588 | *kmt6* | | high | | | N/A | N/A | CCGTCC | 144558218 | 144558218 | 100 |  |
| HTS589 | *kmt6* | | high | | | N/A | N/A | GTCCGC | 186511878 | 186511878 | 100 |  |
