# Supplementary material for: The Fusarium graminearum Histone H3 K27 Methyltransferase KMT6 Regulates Development and Expression of Secondary Metabolite Gene Clusters
Source: PLoS Genet. 2013 Oct 31;9(10):e1003916. doi: 10.1371/journal.pgen.1003916 (PMC3814326; doi:10.1371/journal.pgen.1003916)
Supplement: Table S2 — (DOCX) [file pgen.1003916.s007.docx]

**Supplemental Table 2: Genes included in specific clusters depending on correlation of H3K27me3 enrichment and expression in WT and kmt6.** Only clusters discussed in the manuscript are listed here. Identities of other groups of genes present in specific clusters are available upon request.

| **Locus ID** | **Predicted pfam, known gene name or function** | **Pathway code or cluster name** | **Pathway name or gene name** | **Comments** |
| --- | --- | --- | --- | --- |
| **a. Primary Metabolism clusters 2 and 5 (induced in *kmt6*)** | |  |  |  |
| FGSG_03904 | Glycoside hydrolase, subgroup, catalytic core | BGALACT-PWY | lactose degradation III |  |
| FGSG_00208 | N/A | LIPAS-PWY | triacylglycerol degradation |  |
| FGSG_03194 | Glycoside hydrolase, family 28 | PWY-1081 | homogalacturonan degradation |  |
| FGSG_10615 | N/A | PWY-4441 | DIMBOA-glucoside degradation | also in cluster FG3_40 |
| FGSG_02893 | Protein-arginine deiminase | PWY-4921 | protein citrullination |  |
| FGSG_11326 | Six-hairpin glycosidase | PWY-5767 | glycogen degradation III | sesquiterpencyclase pathway |
| FGSG_08188 | ATPase, P-type, ATPase-associated domain | PWY-6137 | copper transport II |  |
| FGSG_03624 | Concanavalin A-like lectin/glucanase | PWY-6717 | (1,4)-&beta;-xylan degradation |  |
| FGSG_11487 | Glycoside hydrolase, subgroup, catalytic core | PWY-6717 | (1,4)-&beta;-xylan degradation |  |
| FGSG_12047 | N/A | PWY-6717 | (1,4)-&beta;-xylan degradation |  |
| FGSG_12530 | N/A | PWY-6788 | cellulose degradation II (fungi) |  |
| FGSG_07625 | Glycoside hydrolase, family 62, arabinosidase | PWY-6790 | L-arabinan degradation |  |
| FGSG_11036 | Peptidase S9, prolyl oligopeptidase, catalytic domain | PWY-6790 | L-arabinan degradation |  |
| FGSG_09118 | Glycoside hydrolase, subgroup, catalytic core | PWY-6814 | acidification and chitin degradation (in carnivorous plants) | |
| FGSG_02354 | Chitin-binding, type 1 | PWY-6814 | acidification and chitin degradation (in carnivorous plants) | |
| FGSG_03212 | Glycoside hydrolase, subgroup, catalytic core | PWY-6814 | acidification and chitin degradation (in carnivorous plants) | |
| FGSG_07569 | Chitin-binding, type 1 | PWY-6814 | acidification and chitin degradation (in carnivorous plants) | |
| FGSG_01803 | Glycoside hydrolase, clan GH-D | PWY0-1301 | melibiose degradation |  |
| FGSG_00068 | Glycoside hydrolase, clan GH-D | PWY0-1301 | melibiose degradation |  |
| FGSG_11066 | Glycoside hydrolase, subgroup, catalytic core | N/A |  |  |
| FGSG_02347 | Cupin, RmlC-type | TYRFUMCAT-PWY | tyrosine degradation I |  |
| FGSG_00049 | Branched-chain amino acid aminotransferase II | N/A |  |  |
| FGSG_03456 | Alanyl-tRNA synthetase, class IIc, core domain | N/A |  |  |
| FGSG_02314 | Glycoside hydrolase, subgroup, catalytic core | N/A |  |  |
| FGSG_02351 | Carbohydrate-binding domain family 9-like | N/A |  |  |
| FGSG_07921 | Carbohydrate-binding domain family 9-like | N/A |  |  |
| FGSG_08003 | Cellulose-binding domain, fungal | N/A |  |  |
| FGSG_08011 | Cellulose-binding domain, fungal | N/A |  |  |
| FGSG_13826 | Galactose-binding domain-like | N/A |  |  |
| FGSG_02449 | Signal transduction histidine kinase, core | N/A |  |  |
| FGSG_04627 | Signal transduction histidine kinase, core | N/A |  |  |
| FGSG_07812 | Serine/threonine-protein kinase-like domain | N/A |  |  |
| FGSG_07742 | Serine/threonine-protein kinase-like domain | N/A |  |  |
| FGSG_12132 | Serine/threonine-protein kinase-like domain | N/A |  |  |
| FGSG_13509 | Serine/threonine-protein kinase-like domain | N/A |  |  |
| FGSG_09150 | Tyrosine-protein kinase, active site | N/A |  |  |
| FGSG_02450 | N/A | N/A |  |  |
| FGSG_02687 | Glycosyl hydrolase family 43, five-bladed beta-propellor domain | N/A |  |  |
| FGSG_03065 | N/A | N/A | *al-1/carB* |  |
| FGSG_07800 | N/A | N/A | *pks10/fus1* |  |
| FGSG_02399 | N/A | N/A | kinase in zon pathway |  |
| FGSG_03537 | Trichodiene synthase | N/A | *tri5* |  |
| FGSG_03072 | Peptidase S53, propeptide | N/A |  |  |
| FGSG_03562 | Peptidase S58, DmpA | N/A |  |  |
| FGSG_04758 | Peptidase aspartic | N/A |  |  |
| FGSG_04817 | Peptidase S28 | N/A |  |  |
| FGSG_04840 | Peptidase C14, caspase catalytic | N/A |  |  |
| FGSG_05797 | Peptidase S10, serine carboxypeptidase | N/A |  |  |
| FGSG_05815 | Peptidase S8/S53, subtilisin/kexin/sedolisin | N/A |  |  |
| FGSG_01569 | Peptidase C2, calpain, catalytic domain | N/A |  |  |
| FGSG_01818 | Peptidase M28 | N/A |  |  |
| FGSG_02169 | Peptidase M14, carboxypeptidase A | N/A |  |  |
| FGSG_02748 | Peptidase M13 | N/A |  |  |
| FGSG_03027 | Peptidase M28 | N/A |  |  |
| FGSG_08012 | Proteinase inhibitor, propeptide | N/A |  |  |
| FGSG_08022 | Peptidase M28 | N/A |  |  |
| FGSG_11411 | Peptidase M28 | N/A |  |  |
| FGSG_11999 | Peptidase C14, caspase catalytic | N/A |  |  |
| FGSG_03014 | Hexokinase | N/A |  |  |
| FGSG_03284 | N/A | N/A |  |  |
| FGSG_03546 | NAD(P)-binding domain | N/A |  |  |
| FGSG_13963 | NAD(P)-binding domain | N/A |  |  |
| FGSG_07707 | NAD(P)-binding domain | N/A |  |  |
| FGSG_03614 | Alginate lyase domain | N/A |  |  |
| FGSG_03742 | Glucose-methanol-choline oxidoreductase, C-terminal | N/A |  |  |
| FGSG_06444 | Terpenoid synthase | N/A |  |  |
| FGSG_08058 | DNA glycosylase | N/A |  |  |
| FGSG_08852 | N/A | N/A |  |  |
| FGSG_11202 | Guanylate kinase/L-type calcium channel | N/A |  |  |
| FGSG_11429 | N/A | N/A |  |  |
| FGSG_11499 | Glycosyl transferase, family 54 | N/A |  |  |
| FGSG_13790 | N/A | N/A |  |  |
| FGSG_13954 | Fatty acid hydroxylase | N/A |  |  |
| FGSG_00019 | Ras small GTPase, Rab type | N/A |  |  |
| FGSG_02289 | PLC-like phosphodiesterase, TIM beta/alpha-barrel domain | N/A |  |  |
| FGSG_02350 | N/A | N/A |  |  |
| FGSG_03114 | Transferrin receptor-like, dimerisation domain | N/A |  |  |
| FGSG_03129 | N/A | N/A |  |  |
| FGSG_03315 | Proteinase inhibitor, propeptide | N/A |  |  |
| FGSG_03842 | Glycoside hydrolase, subgroup, catalytic core | N/A |  |  |
| FGSG_04604 | Cullin repeat-like-containing domain | N/A |  |  |
| FGSG_04769 | N/A | N/A |  |  |
| FGSG_08139 | Zinc finger, RING-type, conserved site | N/A |  |  |
| FGSG_08141 | N/A | N/A |  |  |
| FGSG_08263 | RelA/SpoT | N/A |  |  |
| FGSG_10595 | Proteinase inhibitor, propeptide | N/A |  |  |
| FGSG_10992 | Glycoside hydrolase/deacetylase, beta/alpha-barrel | N/A |  |  |
| FGSG_11224 | N/A | N/A |  |  |
| FGSG_13519 | Ankyrin repeat-containing domain | N/A |  |  |
| FGSG_13834 | Pectate lyase/Amb allergen | N/A |  |  |
|  |  |  |  |  |
| **b. Primary Metabolism cluster 7 (repressed by high nitrogen)** | |  |  |  |
| FGSG_00173 | N/A | N/A |  |  |
| FGSG_09873 | serine hydroxymethyltransferase, mitochondrial precursor | 1CMET2-PWY |  |  |
| FGSG_02441 | conserved hypothetical protein | BGALACT-PWY |  |  |
| FGSG_09820 | cysteine dioxygenase | CYSTEINE-DEG-PWY |  |  |
| FGSG_07528 | pyruvate kinase | FERMENTATION-PWY |  |  |
| FGSG_08398 | glucosamine-6-phosphate isomerase | GLUAMCAT-PWY |  |  |
| FGSG_01346 | enolase | GLUCONEO-PWY | gluconeogenesis I |  |
| FGSG_03127 | conserved hypothetical protein | GLUCONEO-PWY | gluconeogenesis I |  |
| FGSG_03992 | phosphoglycerate kinase | GLUCONEO-PWY | gluconeogenesis I |  |
| FGSG_05843 | glucose-6-phosphate isomerase | GLUCONEO-PWY | gluconeogenesis I |  |
| FGSG_06055 | hypothetical protein similar to phosphoglycerate mutase | GLUCONEO-PWY | gluconeogenesis I |  |
| FGSG_06257 | glyceraldehyde-3-phosphate dehydrogenase | GLUCONEO-PWY | gluconeogenesis I |  |
| FGSG_00500 | hexokinase | GLUCOSE1PMETAB-PWY | glucose and glucose-1-phosphate degradation |  |
| FGSG_08399 | hexokinase-1 | GLUCOSE1PMETAB-PWY | glucose and glucose-1-phosphate degradation |  |
| FGSG_13651 | conserved hypothetical protein | GLUCOSE1PMETAB-PWY | glucose and glucose-1-phosphate degradation |  |
| FGSG_01433 | glutamate synthase precursor | GLUGLNSYN-PWY |  |  |
| FGSG_07174 | NADP-specific glutamate dehydrogenase | GLUTAMATE-SYN2-PWY |  |  |
| FGSG_09456 | 6-phosphofructokinase | GLYCOLYSIS |  |  |
| FGSG_00175 | 2-methylcitrate synthase, mitochondrial precursor | GLYOXYLATE-BYPASS |  |  |
| FGSG_07313 | hypothetical protein similar to 5-proFAR isomerase | HISTSYN-PWY |  |  |
| FGSG_05912 | hypothetical protein similar to mevalonate kinase | HOMOSER-THRESYN-PWY |  |  |
| FGSG_06645 | hypothetical protein similar to lipid particle protein | LIPAS-PWY |  |  |
| FGSG_02714 | hypothetical protein similar to alpha-1,2-mannosyltransferase Kre5 | MANNOSYL-CHITO-DOLICHOL-BIOSYNTHESIS | |  |
| FGSG_00589 | ribulose-phosphate 3-epimerase | NONOXIPENT-PWY |  |  |
| FGSG_07255 | pyrroline-5-carboxylate reductase | PROSYN-PWY |  |  |
| FGSG_04886 | conserved hypothetical protein | PROUT-PWY |  |  |
| FGSG_09940 | inositol-3-phosphate synthase isozyme 2 | PWY-2301 |  |  |
| FGSG_06822 | glycogen synthase | PWY-5067 |  |  |
| FGSG_06370 | CDP-diacylglycerol-serine O-phosphatidyltransferase | PWY-5669 | phosphatidylethanolamine biosynthesis I |  |
| FGSG_09402 | hypothetical protein similar to sn-1,2-diacylglycerol cholinephosphotransferase | PWY-5669 | phosphatidylethanolamine biosynthesis I |  |
| FGSG_05156 | orotidine 5'-phosphate decarboxylase | PWY-5686 | uridine-5'-phosphate biosynthesis |  |
| FGSG_09678 | hypothetical protein similar to orotate reductase | PWY-5686 | uridine-5'-phosphate biosynthesis |  |
| FGSG_09613 | glycogen phosphorylase | PWY-5941 |  |  |
| FGSG_09845 | hypothetical protein similar to fatty acid desaturase | PWY-5996 |  |  |
| FGSG_02522 | protein SUR2 | PWY-6074 | zymosterol biosynthesis |  |
| FGSG_09830 | C-4 methylsterol oxidase | PWY-6074 | zymosterol biosynthesis |  |
| FGSG_02502 | C-5 sterol desaturase | PWY-6075 | ergosterol biosynthesis |  |
| FGSG_06105 | conserved hypothetical protein | PWY-6100 |  |  |
| FGSG_05023 | glycerol-3-phosphate dehydrogenase | PWY-6118 |  |  |
| FGSG_07916 | conserved hypothetical protein | PWY-6196 |  |  |
| FGSG_05668 | galactose-1-phosphate uridylyltransferase | PWY-6317 |  |  |
| FGSG_02980 | conserved hypothetical protein | PWY-6351 |  |  |
| FGSG_05044 | conserved hypothetical protein | PWY-6352 |  |  |
| FGSG_05154 | hypothetical protein similar to CDP-diacylglycerol-inositol 3-phosphatidyltransferase | PWY-6352 |  |  |
| FGSG_02783 | sterol 24-C-methyltransferase | PWY-6424 |  |  |
| FGSG_07207 | conserved hypothetical protein | PWY-6717 |  |  |
| FGSG_07695 | conserved hypothetical protein | PWY-6717 |  |  |
| FGSG_07892 | conserved hypothetical protein | PWY-6788 |  |  |
| FGSG_00952 | hypothetical protein similar to chitinase 18-3 | PWY-6814 |  |  |
| FGSG_01425 | plasma membrane ATPase | PWY-6814 |  |  |
| FGSG_10049 | cysteine desulfurase, mitochondrial precursor | PWY-6823 |  |  |
| FGSG_04087 | conserved hypothetical protein | PWY3DJ-11470 |  |  |
| FGSG_09476 | conserved hypothetical protein | PWY3O-4106 |  |  |
| FGSG_08706 | hypothetical protein similar to aminoalcoholphosphotransferase | PWY3O-450 |  |  |
| FGSG_01203 | conserved hypothetical protein | PWY66-3 |  |  |
| FGSG_01567 | conserved hypothetical protein | SALVADEHYPOX-PWY |  |  |
| FGSG_08529 | hypothetical protein similar to phosphoserine phosphatase | SERSYN-PWY |  |  |
| FGSG_07926 | hypothetical protein similar to alpha,alpha-trehalose-phosphate synthase subunit TPS2 | TRESYN-PWY |  |  |
| FGSG_06375 | hypothetical protein similar to phospholipid:diacylglycerol acyltransferase | TRIGLSYN-PWY |  |  |
| FGSG_01976 | hypothetical protein similar to aspartate-tRNA ligase | TRNA-CHARGING-PWY |  |  |
| FGSG_02717 | dihydroxy-acid dehydratase | VALSYN-PWY |  |  |
| FGSG_00303 | N/A | N/A |  |  |
| FGSG_00349 | N/A | N/A |  |  |
| FGSG_00397 | N/A | N/A |  |  |
| FGSG_00452 | N/A | N/A |  |  |
| FGSG_00460 | N/A | N/A |  |  |
| FGSG_00469 | N/A | N/A |  |  |
| FGSG_00608 | N/A | N/A |  |  |
| FGSG_00629 | N/A | N/A |  |  |
| FGSG_00639 | N/A | N/A |  |  |
| FGSG_00700 | N/A | N/A |  |  |
| FGSG_00786 | N/A | N/A |  |  |
| FGSG_00797 | N/A | N/A |  |  |
| FGSG_00808 | N/A | N/A |  |  |
| FGSG_00945 | N/A | N/A |  |  |
| FGSG_01028 | N/A | N/A |  |  |
| FGSG_01097 | N/A | N/A |  |  |
| FGSG_01223 | N/A | N/A |  |  |
| FGSG_01231 | N/A | N/A |  |  |
| FGSG_01240 | N/A | N/A |  |  |
| FGSG_01244 | N/A | N/A |  |  |
| FGSG_01300 | N/A | N/A |  |  |
| FGSG_01312 | N/A | N/A |  |  |
| FGSG_01445 | N/A | N/A |  |  |
| FGSG_01501 | N/A | N/A |  |  |
| FGSG_01534 | N/A | N/A |  |  |
| FGSG_01614 | N/A | N/A |  |  |
| FGSG_01618 | N/A | N/A |  |  |
| FGSG_01627 | N/A | N/A |  |  |
| FGSG_01842 | N/A | N/A |  |  |
| FGSG_01898 | N/A | N/A |  |  |
| FGSG_01948 | N/A | N/A |  |  |
| FGSG_02028 | N/A | N/A |  |  |
| FGSG_02067 | N/A | N/A |  |  |
| FGSG_02073 | N/A | N/A |  |  |
| FGSG_02079 | N/A | N/A |  |  |
| FGSG_02262 | N/A | N/A |  |  |
| FGSG_02533 | N/A | N/A |  |  |
| FGSG_02591 | N/A | N/A |  |  |
| FGSG_02706 | N/A | N/A |  |  |
| FGSG_02720 | N/A | N/A |  |  |
| FGSG_03380 | N/A | N/A |  |  |
| FGSG_03418 | N/A | N/A |  |  |
| FGSG_03474 | N/A | N/A |  |  |
| FGSG_03544 | N/A | N/A | i |  |
| FGSG_03582 | N/A | N/A |  |  |
| FGSG_03951 | N/A | N/A |  |  |
| FGSG_03975 | N/A | N/A |  |  |
| FGSG_03978 | N/A | N/A |  |  |
| FGSG_03999 | N/A | N/A |  |  |
| FGSG_04053 | N/A | N/A |  |  |
| FGSG_04068 | N/A | N/A |  |  |
| FGSG_04125 | N/A | N/A |  |  |
| FGSG_04221 | N/A | N/A |  |  |
| FGSG_04289 | N/A | N/A |  |  |
| FGSG_04290 | N/A | N/A |  |  |
| FGSG_04300 | N/A | N/A |  |  |
| FGSG_04364 | N/A | N/A |  |  |
| FGSG_04375 | N/A | N/A |  |  |
| FGSG_04382 | N/A | N/A |  |  |
| FGSG_04418 | N/A | N/A |  |  |
| FGSG_04446 | N/A | N/A |  |  |
| FGSG_04479 | N/A | N/A |  |  |
| FGSG_04505 | N/A | N/A |  |  |
| FGSG_04546 | N/A | N/A |  |  |
| FGSG_04911 | N/A | N/A |  |  |
| FGSG_04980 | N/A | N/A |  |  |
| FGSG_04982 | N/A | N/A |  |  |
| FGSG_05141 | N/A | N/A |  |  |
| FGSG_05164 | N/A | N/A |  |  |
| FGSG_05165 | N/A | N/A |  |  |
| FGSG_05189 | N/A | N/A |  |  |
| FGSG_05306 | N/A | N/A |  |  |
| FGSG_05484 | N/A | N/A |  |  |
| FGSG_05491 | N/A | N/A |  |  |
| FGSG_05514 | N/A | N/A |  |  |
| FGSG_05516 | N/A | N/A |  |  |
| FGSG_05582 | N/A | N/A |  |  |
| FGSG_05586 | N/A | N/A |  |  |
| FGSG_05597 | N/A | N/A |  |  |
| FGSG_05655 | N/A | N/A |  |  |
| FGSG_05697 | N/A | N/A |  |  |
| FGSG_05784 | N/A | N/A |  |  |
| FGSG_05853 | N/A | N/A |  |  |
| FGSG_05902 | N/A | N/A |  |  |
| FGSG_05990 | N/A | N/A |  |  |
| FGSG_06002 | N/A | N/A |  |  |
| FGSG_06053 | N/A | N/A |  |  |
| FGSG_06057 | N/A | N/A |  |  |
| FGSG_06107 | N/A | N/A |  |  |
| FGSG_06117 | N/A | N/A |  |  |
| FGSG_06183 | N/A | N/A |  |  |
| FGSG_06206 | N/A | N/A |  |  |
| FGSG_06209 | N/A | N/A |  |  |
| FGSG_06255 | N/A | N/A |  |  |
| FGSG_06272 | N/A | N/A |  |  |
| FGSG_06297 | N/A | N/A |  |  |
| FGSG_06316 | N/A | N/A |  |  |
| FGSG_06325 | N/A | N/A |  |  |
| FGSG_06527 | N/A | N/A |  |  |
| FGSG_06553 | N/A | N/A |  |  |
| FGSG_06560 | N/A | N/A |  |  |
| FGSG_06572 | N/A | N/A |  |  |
| FGSG_06611 | N/A | N/A |  |  |
| FGSG_06663 | N/A | N/A |  |  |
| FGSG_06793 | N/A | N/A |  |  |
| FGSG_06816 | N/A | N/A |  |  |
| FGSG_06831 | N/A | N/A |  |  |
| FGSG_06832 | N/A | N/A |  |  |
| FGSG_06873 | N/A | N/A |  |  |
| FGSG_06911 | N/A | N/A |  |  |
| FGSG_06923 | N/A | N/A |  |  |
| FGSG_07024 | N/A | N/A |  |  |
| FGSG_07121 | N/A | N/A |  |  |
| FGSG_07151 | N/A | N/A |  |  |
| FGSG_07214 | N/A | N/A |  |  |
| FGSG_07251 | N/A | N/A |  |  |
| FGSG_07332 | N/A | N/A |  |  |
| FGSG_07382 | N/A | N/A |  |  |
| FGSG_07409 | N/A | N/A |  |  |
| FGSG_07473 | N/A | N/A |  |  |
| FGSG_07623 | N/A | N/A |  |  |
| FGSG_07944 | N/A | N/A |  |  |
| FGSG_08031 | N/A | N/A |  |  |
| FGSG_08171 | N/A | N/A |  |  |
| FGSG_08289 | N/A | N/A |  |  |
| FGSG_08298 | N/A | N/A |  |  |
| FGSG_08351 | N/A | N/A |  |  |
| FGSG_08372 | N/A | N/A |  |  |
| FGSG_08415 | N/A | N/A |  |  |
| FGSG_08427 | N/A | N/A |  |  |
| FGSG_08446 | N/A | N/A |  |  |
| FGSG_08454 | N/A | N/A |  |  |
| FGSG_08466 | N/A | N/A |  |  |
| FGSG_08555 | N/A | N/A |  |  |
| FGSG_08583 | N/A | N/A |  |  |
| FGSG_08610 | N/A | N/A |  |  |
| FGSG_08691 | N/A | N/A |  |  |
| FGSG_08731 | N/A | N/A |  |  |
| FGSG_08785 | N/A | N/A |  |  |
| FGSG_08817 | N/A | N/A |  |  |
| FGSG_08846 | N/A | N/A |  |  |
| FGSG_08857 | N/A | N/A |  |  |
| FGSG_08906 | N/A | N/A |  |  |
| FGSG_08962 | N/A | N/A |  |  |
| FGSG_08975 | N/A | N/A |  |  |
| FGSG_08976 | N/A | N/A |  |  |
| FGSG_09226 | N/A | N/A |  |  |
| FGSG_09259 | N/A | N/A |  |  |
| FGSG_09289 | N/A | N/A |  |  |
| FGSG_09612 | N/A | N/A |  |  |
| FGSG_09635 | N/A | N/A |  |  |
| FGSG_09648 | N/A | N/A |  |  |
| FGSG_09725 | N/A | N/A |  |  |
| FGSG_09778 | N/A | N/A |  |  |
| FGSG_09876 | N/A | N/A |  |  |
| FGSG_09897 | N/A | N/A |  |  |
| FGSG_09899 | N/A | N/A |  |  |
| FGSG_09919 | N/A | N/A |  |  |
| FGSG_09920 | N/A | N/A |  |  |
| FGSG_09947 | N/A | N/A |  |  |
| FGSG_10042 | N/A | N/A |  |  |
| FGSG_10066 | N/A | N/A |  |  |
| FGSG_10184 | N/A | N/A |  |  |
| FGSG_10192 | N/A | N/A |  |  |
| FGSG_10201 | N/A | N/A |  |  |
| FGSG_10223 | N/A | N/A |  |  |
| FGSG_10374 | N/A | N/A |  |  |
| FGSG_10387 | N/A | N/A |  |  |
| FGSG_10444 | N/A | N/A |  |  |
| FGSG_10621 | N/A | N/A |  |  |
| FGSG_10659 | N/A | N/A |  |  |
| FGSG_10716 | N/A | N/A |  |  |
| FGSG_10766 | N/A | N/A |  |  |
| FGSG_10800 | N/A | N/A |  |  |
| FGSG_10853 | N/A | N/A |  |  |
| FGSG_10862 | N/A | N/A |  |  |
| FGSG_10873 | N/A | N/A |  |  |
| FGSG_10957 | N/A | N/A |  |  |
| FGSG_11280 | N/A | N/A |  |  |
| FGSG_11588 | N/A | N/A |  |  |
| FGSG_11614 | N/A | N/A |  |  |
| FGSG_12029 | N/A | N/A |  |  |
| FGSG_12866 | N/A | N/A |  |  |
| FGSG_13490 | N/A | N/A |  |  |
|  |  |  |  |  |
| **c. Secondary Metabolites clusters 1, 4 and 7 (induced in *kmt6*)** | |  |  |  |
| FGSG_01736 | N/A | FG3_44 |  |  |
| FGSG_01787 | N/A | fumonisin |  |  |
| FGSG_03064 | carO rhodopsin | carotenoid | carO |  |
| FGSG_03067 | CarX carotenoid dioxygenase | carotenoid | *cao-1/carX* |  |
| FGSG_03534 | trichothecene 15-O-acetyltransferase | trichothecene | *tri3* |  |
| FGSG_03535 | trichodiene oxygenase [cytochrome P450] | trichothecene | *tri4* |  |
| FGSG_03537 | trichodiene synthase [sesquiterpene cyclase] | trichothecene | *tri5* |  |
| FGSG_03538 | regulatory protein | trichothecene | *tri10* |  |
| FGSG_03964 | polyketide synthase | FG3_25 | *pks14/grs1* |  |
| FGSG_04590 | polyketide synthase | FG3_26 | *pks15* |  |
| FGSG_04596 | related to O-methyltransferase | FG3_26 |  |  |
| FGSG_04693 | related to integral membrane protein PTH11 | FG3_1 |  |  |
| FGSG_04694 | polyketide synthase | FG3_1 | *pks2* |  |
| FGSG_05790 | N/A | FG3_12 |  |  |
| FGSG_05794 | polyketide synthase | FG3_12 | *pks5* |  |
| FGSG_07798 | polyketide synthase | Fusarin C | *pks10/fus1* |  |
| FGSG_07800 | related to pepsin A-1 precursor | Fusarin C | *fus4* |  |
| FGSG_07801 | oxidoreductase | Fusarin C | *fus5* |  |
| FGSG_07802 | transporter | Fusarin C | *fus6* |  |
| FGSG_07803 | oxidoreductase | Fusarin C | *fus7* |  |
| FGSG_07804 | cytochrome P450 | Fusarin C | *fus8* |  |
| FGSG_07805 | N/A | Fusarin C |  |  |
| FGSG_08206 | conserved hypothetical protein | FG3_20 |  |  |
| FGSG_08207 | cytochrome P450 | FG3_20 |  |  |
| FGSG_08208 | polyketide synthase | FG3_20 | *pks6* |  |
| FGSG_08209 | non-ribosomal peptide synthetase | FG3_20 | *nps7* |  |
| FGSG_08210 | conserved hypothetical protein | FG3_20 |  |  |
| FGSG_10609 | related to 6-hydroxy-d-nicotine oxidase | FG3_40 |  |  |
| FGSG_10612 | related to salicylate hydroxylase | FG3_40 |  |  |
| FGSG_10614 | N/A | FG3_40 |  |  |
| FGSG_10615 | related to beta-glucosidase precursor | FG3_40 |  |  |
| FGSG_12583 | N/A | FG3_1 |  | next to *pks2* |
| FGSG_13222 | hydrolase | Fusarin C | *fus2* | next to *pks10* |
| FGSG_13223 | translation elongation factor | Fusarin C | *fus3* |  |
| FGSG_13796 | related to Tri13 - putative cytochrome p450 monooxygenase | FG3_40 |  |  |
| FGSG_13797 | related to Tri13 - putative cytochrome p450 monooxygenase | FG3_40 |  |  |
|  |  |  |  |  |
| **d. Secondary Metabolites cluster 8 (repressed in high nitrogen)** | |  |  |  |
| FGSG_02321 | aurO | aurofusarin |  |  |
| FGSG_02324 | aur1(pks-12) | aurofusarin |  |  |
| FGSG_02325 | aurC | aurofusarin |  |  |
| FGSG_02326 | aurJ | aurofusarin |  |  |
| FGSG_02327 | aurF | aurofusarin |  |  |
| FGSG_02328 | gip1 | aurofusarin |  |  |
| FGSG_02329 | aurS | aurofusarin |  |  |
| FGSG_03063 | probable ammonium permease MEPA | carotenoid |  |  |
| FGSG_10547 | related to multidrug resistance protein | FG3_38 |  |  |
| FGSG_10548 | PKS-1 | FG3_38 |  |  |
|  |  |  |  |  |
| **e. Secretome clusters 7 and 8 (induced in *kmt6*)** | |  |  |  |
| FGSG_00060 | N/A | N/A |  |  |
| FGSG_00071 | hypothetical protein similar to cytochrome P450 monooxygenase | PWY66-341 |  |  |
| FGSG_01745 | N/A | N/A |  |  |
| FGSG_02263 | N/A | N/A |  |  |
| FGSG_02309 | N/A | N/A |  |  |
| FGSG_02332 | N/A | N/A |  |  |
| FGSG_02337 | N/A | N/A |  |  |
| FGSG_02354 | hypothetical protein similar to class V chitinase | PWY-6814 |  |  |
| FGSG_02686 | N/A | N/A |  |  |
| FGSG_02893 | conserved hypothetical protein | PWY-4921 |  |  |
| FGSG_03035 | N/A | N/A |  |  |
| FGSG_03129 | N/A | N/A |  |  |
| FGSG_03130 | N/A | N/A |  |  |
| FGSG_03157 | N/A | N/A |  |  |
| FGSG_03194 | polygalacturonase 1 precursor | PWY-1081 |  |  |
| FGSG_03211 | N/A | N/A |  |  |
| FGSG_03212 | conserved hypothetical protein | PWY-6814 |  |  |
| FGSG_03406 | pectinesterase precursor | PWY-1081 |  |  |
| FGSG_03457 | N/A | N/A |  |  |
| FGSG_03550 | N/A | N/A |  |  |
| FGSG_03573 | N/A | N/A |  |  |
| FGSG_03601 | N/A | N/A |  |  |
| FGSG_03700 | N/A | N/A |  |  |
| FGSG_03790 | N/A | N/A |  |  |
| FGSG_03867 | N/A | N/A |  |  |
| FGSG_03883 | N/A | N/A |  |  |
| FGSG_03901 | conserved hypothetical protein | PWY-5344 |  |  |
| FGSG_04563 | N/A | N/A |  |  |
| FGSG_04656 | N/A | N/A |  |  |
| FGSG_04704 | N/A | N/A |  |  |
| FGSG_04741 | N/A | N/A |  |  |
| FGSG_04745 | N/A | N/A |  |  |
| FGSG_04773 | N/A | N/A |  |  |
| FGSG_04793 | N/A | N/A |  |  |
| FGSG_05807 | N/A | N/A |  |  |
| FGSG_05809 | N/A | N/A |  |  |
| FGSG_06479 | N/A | N/A |  |  |
| FGSG_07661 | conserved hypothetical protein | PWY-2841 |  |  |
| FGSG_07921 | N/A | N/A |  |  |
| FGSG_08007 | conserved hypothetical protein | PWY-6481 |  |  |
| FGSG_08012 | N/A | N/A |  |  |
| FGSG_08079 | conserved hypothetical protein | PWY-5034 |  |  |
| FGSG_08210 | N/A | N/A |  |  |
| FGSG_09066 | N/A | N/A |  |  |
| FGSG_09137 | N/A | N/A |  |  |
| FGSG_10435 | N/A | N/A |  |  |
| FGSG_10560 | N/A | N/A |  |  |
| FGSG_10592 | N/A | N/A |  |  |
| FGSG_10615 | hypothetical protein similar to beta-glucosidase | PWY-4441 |  |  |
| FGSG_11008 | N/A | N/A |  |  |
| FGSG_11046 | N/A | N/A |  |  |
| FGSG_11066 | N/A | N/A |  |  |
| FGSG_11101 | N/A | N/A |  |  |
| FGSG_11318 | N/A | N/A |  |  |
| FGSG_11348 | N/A | N/A |  |  |
| FGSG_11436 | N/A | N/A |  |  |
| FGSG_11497 | N/A | N/A |  |  |
| FGSG_11564 | N/A | N/A |  |  |
| FGSG_12816 | N/A | N/A |  |  |
| FGSG_13459 | N/A | N/A |  |  |
| FGSG_13464 | N/A | N/A |  |  |
| FGSG_13834 | N/A | N/A |  |  |
| FGSG_13840 | N/A | N/A |  |  |
| FGSG_13849 | N/A | N/A |  |  |
| FGSG_13952 | N/A | N/A |  |  |
| FGSG_00100 | N/A | N/A |  |  |
| FGSG_02258 | N/A | N/A |  |  |
| FGSG_02314 | N/A | N/A |  |  |
| FGSG_02342 | N/A | N/A |  |  |
| FGSG_02447 | N/A | N/A |  |  |
| FGSG_02685 | N/A | N/A |  |  |
| FGSG_02687 | N/A | N/A |  |  |
| FGSG_02898 | N/A | N/A |  |  |
| FGSG_02910 | N/A | N/A |  |  |
| FGSG_02933 | N/A | N/A |  |  |
| FGSG_03002 | conserved hypothetical protein | PWY-6717 |  |  |
| FGSG_03034 | N/A | N/A |  |  |
| FGSG_03052 | N/A | N/A |  |  |
| FGSG_03121 | N/A | N/A |  |  |
| FGSG_03156 | N/A | N/A |  |  |
| FGSG_03217 | N/A | N/A |  |  |
| FGSG_03236 | N/A | N/A |  |  |
| FGSG_03260 | N/A | N/A |  |  |
| FGSG_03275 | N/A | N/A |  |  |
| FGSG_03304 | N/A | N/A |  |  |
| FGSG_03309 | N/A | N/A |  |  |
| FGSG_03312 | N/A | N/A |  |  |
| FGSG_03402 | N/A | N/A |  |  |
| FGSG_03436 | N/A | N/A |  |  |
| FGSG_03481 | N/A | N/A |  |  |
| FGSG_03531 | N/A | N/A |  |  |
| FGSG_03585 | N/A | N/A |  |  |
| FGSG_03614 | N/A | N/A |  |  |
| FGSG_03624 | endo-1,4-beta-xylanase 2 precursor | PWY-6717 |  |  |
| FGSG_03846 | hypothetical protein similar to T-2 toxin biosynthesis protein | LIPAS-PWY |  |  |
| FGSG_03958 | N/A | N/A |  |  |
| FGSG_03973 | N/A | N/A |  |  |
| FGSG_04614 | N/A | N/A |  |  |
| FGSG_04661 | N/A | N/A |  |  |
| FGSG_04739 | N/A | N/A |  |  |
| FGSG_04740 | N/A | N/A |  |  |
| FGSG_04743 | N/A | N/A |  |  |
| FGSG_04817 | N/A | N/A |  |  |
| FGSG_04841 | N/A | N/A |  |  |
| FGSG_05803 | N/A | N/A |  |  |
| FGSG_06465 | N/A | N/A |  |  |
| FGSG_06466 | N/A | N/A |  |  |
| FGSG_06467 | N/A | N/A |  |  |
| FGSG_07556 | N/A | N/A |  |  |
| FGSG_07562 | N/A | N/A |  |  |
| FGSG_07625 | alpha-L-arabinofuranosidase precursor | PWY-6790 |  |  |
| FGSG_07670 | N/A | N/A |  |  |
| FGSG_07671 | N/A | N/A |  |  |
| FGSG_07684 | N/A | N/A |  |  |
| FGSG_07714 | N/A | N/A |  |  |
| FGSG_07728 | N/A | N/A |  |  |
| FGSG_07808 | N/A | N/A |  |  |
| FGSG_07972 | N/A | N/A |  |  |
| FGSG_07981 | N/A | N/A |  |  |
| FGSG_07988 | N/A | N/A |  |  |
| FGSG_08002 | N/A | N/A |  |  |
| FGSG_08011 | N/A | N/A |  |  |
| FGSG_08015 | N/A | N/A |  |  |
| FGSG_08023 | N/A | N/A |  |  |
| FGSG_08026 | N/A | N/A |  |  |
| FGSG_09071 | N/A | N/A |  |  |
| FGSG_09093 | N/A | N/A |  |  |
| FGSG_09098 | N/A | N/A |  |  |
| FGSG_09118 | conserved hypothetical protein | PWY-6814 |  |  |
| FGSG_09353 | N/A | N/A |  |  |
| FGSG_10451 | benzoate 4-monooxygenase | PWY-5034 |  |  |
| FGSG_10554 | N/A | N/A |  |  |
| FGSG_10585 | N/A | N/A |  |  |
| FGSG_10609 | N/A | N/A |  |  |
| FGSG_10670 | N/A | N/A |  |  |
| FGSG_10695 | conserved hypothetical protein | PWY-5034 |  |  |
| FGSG_11006 | N/A | N/A |  |  |
| FGSG_11036 | conserved hypothetical protein | PWY-6790 |  |  |
| FGSG_11077 | N/A | N/A |  |  |
| FGSG_11078 | conserved hypothetical protein | PWY-5686 |  |  |
| FGSG_11100 | conserved hypothetical protein | PWY-5418 |  |  |
| FGSG_11156 | N/A | N/A |  |  |
| FGSG_11191 | N/A | N/A |  |  |
| FGSG_11204 | N/A | N/A |  |  |
| FGSG_11208 | N/A | N/A |  |  |
| FGSG_11227 | N/A | N/A |  |  |
| FGSG_11232 | N/A | N/A |  |  |
| FGSG_11238 | N/A | N/A |  |  |
| FGSG_11257 | conserved hypothetical protein | PWY-5686 |  |  |
| FGSG_11276 | N/A | N/A |  |  |
| FGSG_11315 | N/A | N/A |  |  |
| FGSG_11361 | N/A | N/A |  |  |
| FGSG_11373 | N/A | N/A |  |  |
| FGSG_11379 | N/A | N/A |  |  |
| FGSG_11487 | endo-1,4-beta-xylanase precursor | PWY-6717 |  |  |
| FGSG_11496 | N/A | N/A |  |  |
| FGSG_12067 | N/A | N/A |  |  |
| FGSG_12123 | N/A | N/A |  |  |
| FGSG_12434 | N/A | N/A |  |  |
| FGSG_12445 | N/A | N/A |  |  |
| FGSG_12548 | N/A | N/A |  |  |
| FGSG_12591 | N/A | N/A |  |  |
| FGSG_12644 | N/A | N/A |  |  |
| FGSG_12918 | N/A | N/A |  |  |
| FGSG_13219 | N/A | N/A |  |  |
| FGSG_13450 | N/A | N/A |  |  |
| FGSG_13462 | N/A | N/A |  |  |
| FGSG_13958 | N/A | N/A |  |  |
| FGSG_13963 | N/A | N/A |  |  |
|  |  |  |  |  |
| **f. Secretome cluster 9 (repressed in high nitrogen)** | |  |  |  |
| FGSG_02327 | N/A | *aurF* |  |  |
| FGSG_02328 | hypothetical protein similar to brown 2 | *gip1* |  |  |
| FGSG_02329 | N/A | *aur* cluster unnamed gene |  |  |
| FGSG_06993 | N/A | N/A |  |  |
| FGSG_11125 | N/A | N/A |  |  |
| FGSG_11164 | N/A | N/A |  |  |
| FGSG_11249 | N/A | N/A |  |  |
| FGSG_12207 | N/A | N/A |  |  |
